# A Dress Is (Still) Not a Yes: How Even Simple Key Presses Reveal Sexually Aroused Men’s Overreliance on Global Cues in the Context of Sexual Flirting

**DOI:** 10.1007/s10508-026-03449-7

**Published:** 2026-06-01

**Authors:** Ingo Landwehr, Katrin Landwehr, Alexander F. Schmidt

**Affiliations:** 1 Institute of Psychology, Social and Legal Psychology, Johannes Gutenberg University Mainz, Binger Str. 14–16, 55122 Mainz, Germany; 2 Nuremberg, Germany

**Keywords:** Sexual arousal, Sexual decision-making, Flirting, Cue weighting, Key-press paradigm

## Abstract

In a pilot study using mouse-tracking, we examined men’s interpretation and weighting of global sexual cues (e.g., clothing style) and specific affective cues (e.g., facial expression) when judging women’s flirting intentions. Sexual arousal systematically biased this assessment by increasing the likelihood of overreliance on global cues (OGC). Error rate (ER) emerged as a behavioral indicator of OGC, whereas reaction time (RT) captured complementary information on decision dynamics. Notably, ER also showed a theoretically meaningful pattern of positive correlations with self-report trait measures of interpersonally problematic sexuality (e.g., disinhibited, exploitative). We sought to replicate these findings in a substantially larger sample, using a simplified key-press paradigm and focusing solely on RT and ER as dependent variables. In a preregistered online study with heterosexual men (*N* = 284), experimentally induced sexual arousal again increased the likelihood of OGC in a sexual flirting task. The critical main and interaction effects were replicated and a similar pattern of positive correlations emerged between the experimental measures and self-report measures of dispositional risk factors for sexually problematic behavior. However, a somewhat different moderation pattern by these individual differences was observed. Taken together, the simplification of the paradigm proved largely successful and the resulting approach appears promising for diagnostic applications in forensic and clinical contexts.

**Supplementary Information:**

The online version contains supplementary material available at 10.1007/s10508-026-03449-7.

## Introduction

From a social-normative perspective, several evaluative decisions must be made prior to sexually motivated approaches. While the assessment of others based on their sexual attractiveness—i.e., answering the question “Do I want to approach?”—occurs largely automatically (Janssen et al., 2000), the consideration of social appropriateness—i.e., answering the question “May I approach?”—requires a reflective evaluation of others’ intentions, which are not readily discernible (Epley & Kardas, 2021). Unsurprisingly, this evaluation can result in misjudgments and misunderstandings (Perilloux, 2014; Perilloux et al., 2012), potentially leading to serious consequences for those involved. The so-called overperception bias describes the tendency of men to overestimate women’s sexual interest (Bondurant & Donat, 1999; Haselton, 2003), which is particularly likely when less informative non-affective cues are used to assess sexual intent, while more informative affective cues are disregarded (Smith et al., 2018). Previous research has shown that this bias varies greatly among men (Jacques-Tiura et al., 2007) and is associated with various socially and sexually problematic beliefs and behaviors, including hypermasculinity (Fisher & Walters, 2003), rape-supportive attitudes (Treat et al., 2020), sexual coercion (Farris et al., 2008), and sexual aggression (Abbey, 1991). Additionally, various situational factors have been found to contribute to an increased likelihood of men misinterpreting social and sexual signals displayed by women, including alcohol intoxication (Abbey et al., 1996, 2000), sexual arousal (Rerick et al., 2020), and cue incongruence (Farris et al., 2010b).

### Flirting and Its Cues

To respond appropriately to the intentions of others in social settings (i.e., to avoid socially undesirable behavior and its consequences), it is essential to correctly interpret and weight the informative cues displayed by another person. Informative cues vary in nature and can be categorized as verbal, visual, haptic, or paralinguistic (Fichten et al., 1992). During the early stages of potential flirting, particularly when individuals are spatially separated and no direct interaction has yet occurred, the evaluation of a person’s intent typically relies on visual cues. From a heterosexual man’s perspective, a woman’s more dynamic and context-sensitive cues—i.e., cues directed specifically at the evaluating man—are particularly informative, in contrast to more general cues that can be perceived by various individuals across different situations and over time. The latter, referred to as “omni-directional” (Treat et al., 2016b) or “global” (Landwehr et al., 2024) cues, include clothing style/attire, physical attractiveness, and, to some extent, general demeanor. Such cues are cross-situationally stable and therefore less indicative of a woman’s response to a specific man at a given moment in time, provided that the woman is in an open social space (e.g., a club) and has not arranged to meet a particular man (in which case her attire might indeed carry specific informational value). Conversely, affective cues such as facial expression and body language are more “uni-directional” (Treat et al., 2016b) and can be directed at a specific man in a particular situation (hence referred to as “specific” cues by us; Landwehr et al., 2024), with deliberate eye contact, especially when mutual, serving as a strong indicator of the intended recipient of these signals (Adams & Kleck, 2005).

Global and specific cues thus clearly differ in the type of information they can convey in a dyadic social interaction, as specific cues are inherently tied to that interaction, whereas global cues are not and may at most reflect a personal preference, general intention, or mood. Neglecting this difference in informational value within the context of flirting increases the risk of overreliance on global cues (OGC), meaning that inadequate (i.e., nonspecific) information is primarily used to evaluate another person’s sexual interest. Such misweighting is particularly likely when top-down expectations or goals (e.g., sexual contact) interfere with conflicting bottom-up information (e.g., a facial expression indicative of sexual disinterest) (Freeman & Johnson, 2016), consistent with general models of confirmatory perception (Oeberst & Imhoff, 2023).

It has been demonstrated that non-affective cues displayed by women, such as their clothing style and attractiveness, particularly distort the perception of their sexual interest among men at greater risk of committing sexual aggression (Treat et al., 2016a). Similarly, Farris et al. (2010a) observed a reduction in men’s ability to detect dating-relevant affective cues in full-body photos of women when the women were dressed in more revealing attire, with the influence of clothing style on judgments about women’s sexual interest being significantly greater among men endorsing rape-supportive attitudes. Building on such findings, Treat et al. (2011) concluded that provocative clothing and attractiveness may act as distractors, leading to a diminished ability to process affective cues related to sexual interest and rejection. Since such a diminished ability to correctly interpret and weight sexual (dis)interest cues represents a significant risk factor for sexually offensive behavior (e.g., Racey et al., 2000), Abbey et al. (2011) extended Malamuth et al.’s (1991) confluence model by incorporating the misperception of women’s sexual intent as a proximal predictor of sexual aggression.

### Sexual Arousal

Experimental evidence on how visceral states influence information processing comes from various studies that explored their effects on perception, attention, and cognitive accessibility (Seibt et al., 2007). Selection tasks, in particular, have shown that visceral states influence decision-making and choice behavior by creating a motivational preference for stimuli that promise faster or more complete alleviation of currently experienced deprivation and, as a result, greater immediate satisfaction (de Ridder et al., 2014). As has been demonstrated, this effect also applies to sexual arousal (Ariely & Loewenstein, 2006), which increases the appeal of sexual stimuli through the anticipation of sexual pleasure (Meston & Buss, 2007). Therefore, it is likely that sexually aroused individuals focus their attention on cues that are perceived to align with their current motivational state and offer the prospect of gratification, rather than fully processing all available information, including disconfirming cues, in a given decision-making situation (Loewenstein, 1996).

Consequently, sexually aroused men are more susceptible to misunderstandings in sexual communication (Rerick et al., 2020), particularly because they tend to perceive women as more sexually attractive (Ditto et al., 2006) and as sexually aroused themselves (Maner et al., 2005), and to overestimate their sexual willingness (Livingston et al., 2022). Furthermore, when sexually aroused, men are more prone to exhibit sexually disinhibited behavior (Imhoff & Schmidt, 2014; Wiemer et al., 2023) and engage in sexual risk-taking (Skakoon-Sparling et al., 2016).^1^ Notably, this pattern persists even in contexts where restraining sexual behavior is socially expected, whereas acting on sexual impulses is met with sanctions (Bouffard, 2011). For instance, experimental studies have shown that acute sexual arousal increases men’s self-reported likelihood of sexually aggressive behavior (Craig et al., 2020; Davis et al., 2006; Loewenstein et al., 1997). In line with this, Bouffard and Miller (2014) found that sexual arousal was associated with a higher likelihood of sexual coercion, in part resulting from a distorted perception of the victim’s sexual intent.

### Current Study

In a previous study using mouse-tracking (Landwehr et al., 2024), we demonstrated that sexual arousal increases the likelihood of OGC, which was assessed under cue incongruence (i.e., a condition in which specific affective and global sexual cues convey differing information). However, since the spatial mouse-tracking measures of maximum deviation and area under the curve proved less informative than the more conventional measures of reaction time (RT) and error rate (ER), we ultimately posed the question of whether similar results could be obtained using a purely key-press–based design. Accordingly, revised hypotheses and a streamlined study design were preregistered prior to data collection (https://aspredicted.org/hjq8-mttr.pdf). The key-based paradigm promised several pragmatic advantages, including a reduction in experimental parameters, simpler statistical analyses, and more straightforward interpretation of the results. This approach seemed particularly justified in light of findings on RT measurement, suggesting that there are no functional differences between conflict tasks conducted using a computer mouse and those using key presses (De-Marchis, 2013).

#### Flirting Task Rationale

As in the prior mouse-tracking study (Landwehr et al., 2024), we built on extensive theoretical and empirical groundwork from previous research on the (mis)perception of sexual (dis)interest (e.g., Smith et al., 2018; Treat et al., 2020). We extended this work by incorporating (1) two distinct trial types and (2) two measurements (approximately 15 min apart), with sexual arousal induced in between. Instead of asking participants to assign a single pictorial stimulus to one of two competing categories (e.g., Townsend et al., 2000), we again used a two-choice design in which participants selected the image they considered the correct response to the given instruction. This approach aligns with Loewenstein’s (1996) criteria for the influence of visceral states on decision-making, namely the temporal proximity of the decision-maker’s visceral state to the decision and the presence of sensory (e.g., visual) input indicating physical proximity to desired objects. The corresponding design allowed us to examine whether men, when sexually aroused, prioritize global sexual cues over specific affective ones when assessing a woman’s flirtation intent, despite their objectively lower informational value in dyadic social interactions. To this end, we employed a task in which participants had to decide which of two concurrently displayed images depicted a woman as more likely to be flirting with them, i.e., indicating sexual interest.^2^ Since both images in a given trial depicted the same woman, the experimental design ensured systematic variation in only one global sexual cue (i.e., attire) and one specific affective cue (i.e., facial expression), leaving these cues as the sole sources of diverging information available for participants’ judgments.

By systematically combining these global and specific cues, it was also possible to create two distinct trial types: one consisting of two images with stereotypically congruent cue combinations and the other consisting of two images with stereotypically incongruent cue combinations (see Stimuli and Trial Types section for details). Drawing on previous research demonstrating that incongruent trials increase decision difficulty (e.g., Landwehr et al., 2024; Smith et al., 2018), we expected congruency effects in the present study, with participants requiring more time and making more errors in incongruent trials compared to less demanding congruent ones. The clear contrast between the two displayed facial expressions (see Stimuli and Trial Types section) ensured that the task captured only genuine categorization errors, meaning that misinterpretations within the same cue category (e.g., mistaking friendliness for sexual interest) due to ambiguous expressions were not experimentally considered (for studies on differentiation errors caused by cue ambiguity, see Abbey, 1987; Shotland & Craig, 1988). We extended our original hypothesis on the effect of incongruent trials by adding a correlational hypothesis, stating that a higher degree of selection conflict would both slow down the decision-making process and impair response accuracy at the trial level. For this purpose, we applied the same rationale as in our previous study (Landwehr et al., 2024), expecting trials consisting of images with similar levels of sexual attractiveness (i.e., higher cue similarity) to elicit greater decisional uncertainty.

Given earlier findings on the effects of sexual arousal, it appeared important to further examine whether the “motivational myopia” (Ditto et al., 2006) induced by sexual arousal influences men’s cue-based evaluation of potential sexual partners “in the heat of the moment” (Ariely & Loewenstein, 2006). Specifically, we tested whether, and to what extent, sexually aroused men are prone to ignoring discrepancies between their own and a woman’s sexual intent by relying on global sexual rather than specific affective cues in a flirting context, and whether men with a propensity toward OGC exhibit characteristics that systematically distinguish them from the rest of the sample. In our earlier study (Landwehr et al., 2024), we hypothesized that a state of sexual arousal would make it more difficult for participants to choose between the two response options, regardless of trial type. Accordingly, we assumed that both RT and ER would increase compared to a non-arousal condition. Instead, we found that RTs at the second measurement (T2) were significantly shorter than at the first (T1). A review of the literature revealed that Benbouriche et al. (2019) reported a similar, likewise unexpected, decrease in RT during sexual arousal. As a possible explanation for this phenomenon, Benbouriche et al. (2019) suggested either a priming effect (Collins & Loftus, 1975) or an impression management effect (Paulhus, 2002). They also considered that participants, having recognized the experimental objectives, may have exhibited heightened attentional control following the induction of sexual arousal (Benbouriche et al., 2019). This latter explanation seemed questionable to us, given the increased ERs in the sexual arousal condition observed in our own study (Landwehr et al., 2024). We therefore emphasized the disinhibitory effect of sexual arousal documented in previous studies (Imhoff & Schmidt, 2014; Wiemer et al., 2023), which could promote automated and thus (1) faster and (2) more error-prone responses, particularly when need-congruent stimuli are present.

Sexual arousal was again induced between T1 and T2 in the present study. This setup ensured that the baseline condition at T1 was completed in a comparatively low-arousal (i.e., “cold” visceral) state, allowing us to observe the potential impact of “hot” states on decision-making (Ariely & Loewenstein, 2006; Skakoon-Sparling et al., 2016) at T2 relative to T1. We assumed that making the correct choice (i.e., one that aligns with the instruction) between the two female stimuli would generally be more difficult in a state of sexual arousal at T2 than in the baseline condition at T1. We also expected this difficulty to increase further under higher selection conflict, that is, when in a state of sexual arousal, specific affective cues had to be considered for a correct choice, even if they were presented in a stereotypically incongruent manner with global sexual cues. However, the consistency between our earlier findings and those of Benbouriche et al. (2019), along with the plausible assumption of more automatic response behavior under arousal, led us to revise our original hypotheses, such that we now expected sexual arousal to accelerate decision-making in the selection task rather than to decelerate it. Specifically, because RTs in congruent (low-conflict; LC) trials were assumed to approach a performance floor already under baseline conditions, whereas incongruent (high-conflict; HC) trials were assumed to impose greater inhibitory demands due to competing cue information, we hypothesized that arousal-related facilitation effects from T1 to T2 would be more pronounced in HC than in LC trials. In addition, we expected this facilitative effect to be stronger in the first half of T2 than in the second half due to a gradual decrease in sexual arousal over time, consistent with the transient nature of arousal states.

The everyday relevance of the paradigm was ensured not only at the construct level but also through its thematic embedding. We again employed a flirting framework for our selection task to leverage a common real-world scenario frequently associated with sexual motivation (Moore, 2010). Sexual arousal was noninvasively induced through an erotic audio narrative, i.e., without relying on explicit visual content. As shown by Both et al. (2011), feelings of sexual arousal are enhanced when participants adopt an emotion-oriented focus rather than a stimulus-oriented focus while viewing erotic stimuli. It can be assumed that our task elicited such an emotion-oriented focus by conveying a sense of meaningful social interaction, as each depicted woman made eye contact with the observer, thereby addressing him directly. The instruction for the flirting task (i.e., “Click on the woman who is more likely to flirt with you right now”) was designed to emphasize the perceived sexual interest of the depicted women, while participants’ own sexual interest was deliberately excluded, rendering it conceptually irrelevant to the decision.

### Individual Differences Perspective

According to our reasoning, alterations in both RT and ER may serve as behavioral indicators of an individual’s susceptibility to OGC and its potential real-world consequences, including socially inappropriate or non-consensual sexual advances. From a theoretical standpoint, such interpretations would be particularly informative if OGC-related performance patterns were to covary with stable individual differences that reflect problematic (e.g., manipulative, exploitative, and disinhibited) sexual attitudes, behaviors, and motivations (i.e., “problematic sexuality”), antagonistic personality traits (i.e., the Dark Triad; Lyons et al., 2020), or a reduced sensitivity to social norms.

Building on this theoretical perspective, the present study examined whether OGC-related performance indices are associated with, and moderated by, individual differences in (1) sex drive, (2) sexual objectification, (3) sexual narcissism, (4) Dark Triad traits, and (5) propensity for socially deviant behavior. Sex drive and sexual objectification were already examined in our previous mouse-tracking study and were included again to assess the robustness of these effects across paradigms. Sexual narcissism, Dark Triad traits, and propensity for socially deviant behavior were newly introduced to extend the individual differences perspective. The theoretical rationales for sex drive and sexual objectification are reported in the prior study (Landwehr et al., 2024); detailed rationales for the newly included moderators are provided in the Electronic Supplement (ES1).

#### Hypotheses

Based on the presented theoretical rationale and study design, we preregistered (https://aspredicted.org/hjq8-mttr.pdf) the following hypotheses:

- H1. In HC trials, (a) RTs will be longer and (b) ERs higher than in LC trials (i.e., main effect of trial type).
- H2. At T2, (a) RTs will be shorter and (b) ERs higher than at T1 (i.e., main effect of time).
- H3. The influence of sexual arousal, as described in H2, will be more pronounced in HC trials than in LC trials (i.e., interaction between trial type and time).
- H4. The strength of the interaction described in H3 will increase with higher levels of (a) sex drive, (b) sexual objectification, (c) sexual narcissism, (d) Dark Triad traits, and (e) past socially deviant behavior (i.e., moderation effects).
- H5. In HC trials, greater conflict potential (i.e., higher cue similarity) will correlate with (a) longer RTs and (b) higher ERs.
- H6. Assuming a decrease in sexual arousal over the course of T2, the interaction effects described in H3 will be more pronounced in the first half of T2 than in the second half.
- H7. A consistent pattern of positive correlations will emerge between RT and ER difference scores (see Statistical Analyses section) and measures of (a) problematic sexuality, (b) Dark Triad traits, and (c) past socially deviant behavior.

## Method

### Participants

Initially, the convenience sample consisted of 314 adult German-speaking participants recruited via the online panel provider Prolific. Eligibility criteria required participants to self-identify as (1) male and (2) exclusively or predominantly heterosexual, both of which were verified through Prolific’s pre-screening filters and explicit in-study items. Consistent with the preregistration, participants were excluded from further analyses based on the following criteria: (1) identifying as female (*n* = 2) or omitting key demographic information, thereby preventing verification of eligibility (*n* = 1); (2) reporting a Kinsey score (Kinsey et al., 1948) above 2 (*n* = 6), indicating insufficient sexual attraction to women; (3) providing an honesty rating below 4 on a 5-point scale (*n* = 1); and (4) indicating that they had not listened to the full audio stimulus (*n* = 14), precluding the assumption of sexual arousal at T2. Next, data were screened for participant-level outliers using Tukey’s criterion (> 3 IQR). While (5) no outliers were detected for RT, (6) six participants showed exceptionally low response rates (i.e., trials with missing responses), likely reflecting low task engagement or technical issues, and were thus excluded. ER was exempt from outlier screening, as high individual ERs were considered theoretically meaningful. After applying all exclusion criteria, the final sample comprised *N* = 284 men.

Participants’ ages ranged from 18 to 73 years (*M* = 29.86, *SD* = 9.04). The majority (87%) identified as exclusively heterosexual (Kinsey level 0), while the remainder reported occasional same-sex fantasies or experiences (11% at level 1, 2% at level 2). Most participants (61%) reported being in a committed relationship. Regarding educational attainment, 51% reported holding a university degree, 37% a general higher education entrance qualification (“Abitur”), 10% an intermediate secondary school certificate (“Realschulabschluss”), and 2% a lower secondary school certificate (“Hauptschulabschluss”). One participant reported having no formal educational qualification.

### Measures

#### Stimuli and Trial Types

As in the previous mouse-tracking study (Landwehr et al., 2024), the flirt decision task in the current study drew on a stimulus set of 384 pictorial stimuli (i.e., full-body photographs of adult women), which had been specifically developed and validated through a prior multi-step procedure (Landwehr & Landwehr, 2025; for details on the creation and validation process, see ES2). The images systematically varied along two key dimensions: (1) facial expression (flirtatious/interested vs. rejecting/dismissive) and (2) clothing style (casual/unobtrusive vs. revealing/provocative). Each image had been evaluated by *N* = 150 raters from an age-stratified sample according to three criteria: (1) the effect of facial expression, (2) the effect of clothing, and (3) perceived sexual attractiveness. The stimuli were characterized by high ecological validity, as all models brought their own authentic attire (e.g., clothing, accessories, and makeup) to the photo shoots, i.e., outfits they would actually wear in social situations resembling the experimental conditions: (1) “visiting a good friend in casual clothing to hang out” and (2) “going out at night in a sexy outfit with the general intention to flirt.” Additionally, the models were instructed to display affective facial expressions corresponding to two imagined (yet familiar) social situations in which they would either (1) “flirt with a person perceived as sexually attractive” or (2) “unequivocally reject an obnoxious suitor.” Since the images had been standardized in terms of various aspects (e.g., body posture, lighting, and final editing), potential experimental effects could clearly be attributed to variations in global (clothing) and specific (facial expression) cue combinations. For the current study, a subset of these stimuli, consisting of 208 images (plus 12 practice-trial images), was used.

We again employed a selection task in which participants chose between two simultaneously presented images of the same woman. The main advantage of such a two-image design lies in the fact that (1) selection conflict arises directly from competing visual stimuli, without requiring image-to-text abstraction. Moreover, (2) decisions are based on a relative instruction (“Click on the woman who is more likely to flirt with you right now”) rather than on absolute binary categories, thereby (3) avoiding the explicit introduction of rejection and allowing for more naturalistic judgments. Crucially, this design also (4) prevents confounds arising from subjectively perceived differences in overall sexual attractiveness between women (cf. Rerick & Livingston, 2022, for a contrasting approach). Finally, since the two images of a trial differ only with respect to attire and facial expression cues, the task can be considered relatively easy to solve, thus (5) highlighting the diagnostic relevance of instruction-inconsistent responses as an indicator of OGC.

Standardized cue variations in the images used allowed us to systematically combine stimuli to create trial types that would elicit varying levels of decision difficulty. Accordingly, in the incongruent HC condition, one image showed a woman in casual everyday clothing with a flirtatious facial expression (Casual × Flirting combination), whereas the other image showed her in sexy clothing with a rejecting facial expression (Sexy × Rejecting combination). In contrast, in the congruent LC condition, one image showed a woman with both cues consistent with sexual interest (Sexy × Flirting combination), while the other image showed her with both cues not consistent with sexual interest (Casual × Rejecting combination). In line with the previous study the previous study (Landwehr et al., 2024), we selected image pairs expected to exhibit either relatively relatively high conflict potential (i.e., HC pairs in which the conceptually correct and incorrect images were rated similarly in sexual attractiveness) or relatively relatively low conflict potential (i.e., LC pairs in which the conceptually correct image was rated higher in sexual attractiveness). The images were divided into two parallel sets (each stimulus was presented only once), matched for mean and distributed sexual attractiveness values (for details on the stimuli and parallel sets used in this study, see ES3).

**Fig. 1:**
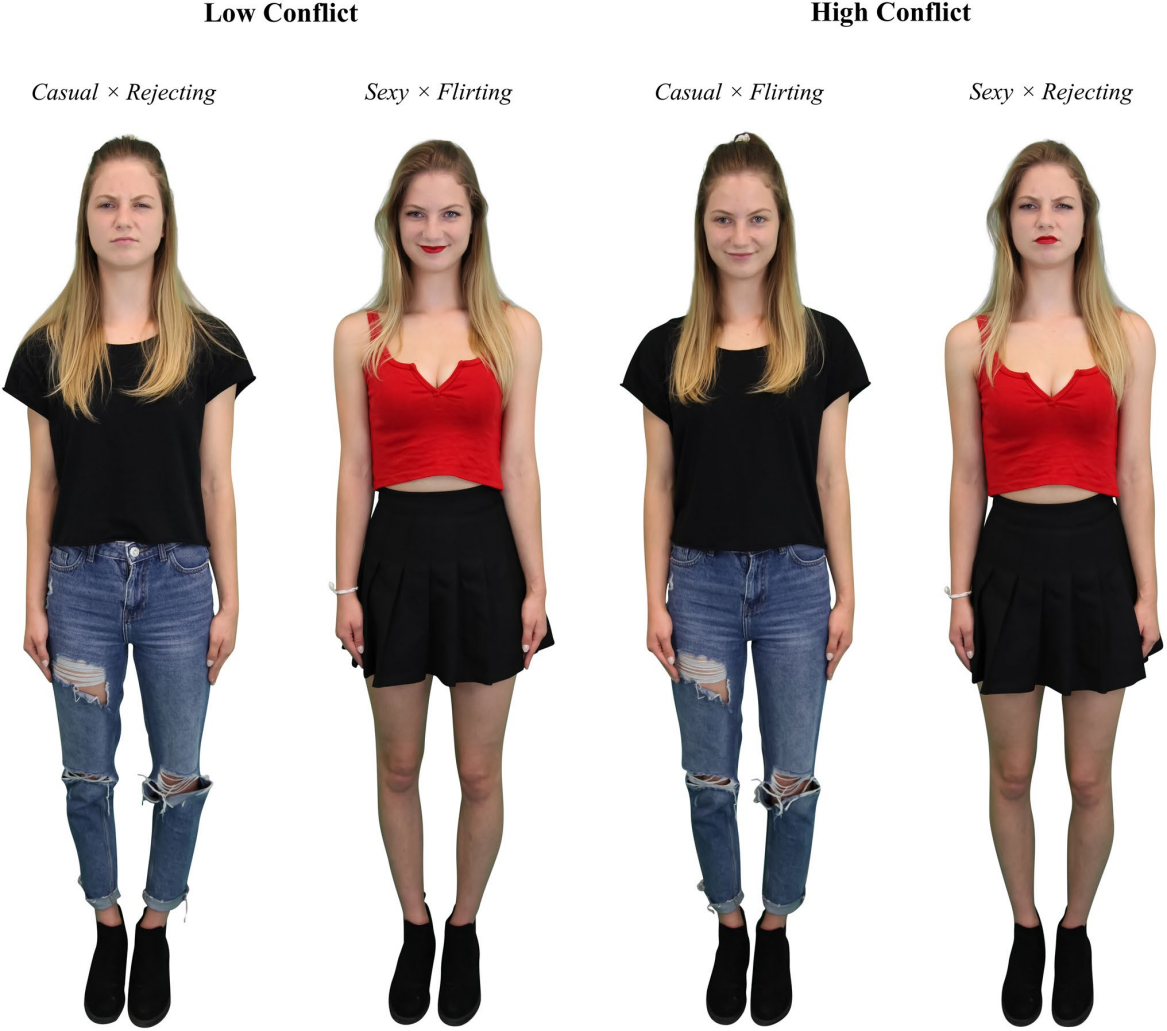
Trial types and cue combinations

The juxtaposition in HC trials was based on the assumption that both a flirtatious facial expression and a sexually suggestive outfit are cues that men perceive as sexually appealing, thus facilitating male sexual selection (e.g., Grammer et al., 2004; Walsh & Hewitt, 1985). At the same time, it was reasonable to assume that both a rejecting facial expression and unobtrusive clothing would not be perceived as equally positive. Instead, these cues were expected to be perceived as rather neutral (in the case of the casual outfit) or even negative (in the case of the rejecting facial expression) and were therefore assumed not to facilitate, but rather to inhibit, male sexual selection (e.g., Goodboy & Brann, 2010; Moore, 1998). Since in the HC condition both images were incongruent, i.e., each contained one positive and one non-positive cue, a prompt to choose between them could be expected to evoke a certain degree of selection conflict. In contrast, in LC trials, when confronted with two juxtaposed congruent images, participants’ choices were expected to clearly favor the image displaying the Sexy × Flirting combination, as this combination would correspond to both the more sexually appealing option (regarding clothing) and the conceptually correct affective option (regarding facial expression). Figure 1 depicts example trials from the HC and LC conditions along with their respective cue combinations.

#### Questionnaires

We employed nine self-report questionnaires to assess individual differences in potentially problematic sexuality, Dark Triad traits, and past socially deviant behavior.^3^ Questionnaires were presented in a fixed, pre-randomized order to maximize between-subjects variance. To minimize participant fatigue, validated short forms were used when available. Sexual desire was measured with the Sexual Desire Inventory-2 (SDI-2; Spector et al., 1996; German short version Kuhn et al., 2014; ten 8-point items, two items from 1 = *never* to 8 = *more than once a day*, five items from 1 = *no desire* to 8 = *strong desire*, two items from 1 = *much less desire* to 8 = *much more desire*, one item from 1 = *unimportant* to 8 = *extremely important*). The scale comprises two subscales (Dyadic Sexual Desire and Solitary Sexual Desire), whose scores can be combined into a total score. Individual differences in sexual excitation were assessed with the corresponding subscale of the Sexual Inhibition/Sexual Excitation Scales (SIS/SES; Janssen et al., 2002; German short version Turner et al., 2014; six 5-point items from 1 = *does not apply at all* to 5 = *applies completely*). The revised Sociosexual Orientation Inventory (SOI-R; Penke & Asendorpf, 2008; nine 5-point items, three items from 1 = 0 to 5 = *8 or more*, three items from 1 = *I do not agree at all* to 5 = *I agree completely*, three items from 1 = *never* to 5 = *almost every day*) measures individual preferences for impersonal sex and casual sexual encounters. Because SDI-2, SES, and SOI-R represent distinct but typically highly intercorrelated facets of sex drive, we calculated a composite score of their *z*-standardized values—referred to as the Sex Drive Composite Index (SDCI)—to measure sex drive on a higher level of aggregation, as in Landwehr et al. (2024). A tendency toward sexual narcissism was assessed using the two subscales Sexual Entitlement and Low Sexual Empathy of the Sexual Narcissism Scale (SNS; Widman & McNulty, 2010; German version Imhoff et al., 2013; ten 5-point items from 1 = *does not apply at all* to 5 = *applies completely*).^4^ The Sexual Objectification of Others Inventory (SOOI; Anslinger, 2019; ten 5-point items from 1 = *strongly disagree* to 5 = *strongly agree*) was used to assess the propensity to perceive women primarily in terms of their sexual appeal and functionality, based on the two factors Instrumental Objectification and Visual Objectification. Participants also answered the Socially Undesirable Sexual Selection Scale (SUSS; Landwehr et al., 2024; 13 items to be answered on a visual analog scale from 0 = *very unlikely* to 100 = *most likely*). This unidimensional scale was developed to capture indications of manipulative flirting behavior and includes items with high face validity, ensuring that their relevance to the research question is apparent (example item: “I am willing to deceive the woman so that she will have sex with me”). We expected that willingness to use manipulative flirting tactics would correlate positively with OGC, since both are driven by the man’s egocentric sexual interest rather than a consideration of the woman’s interest. Self-perceived mate value was assessed via the Mate Value Scale (MVS; Edlund & Sagarin, 2014; German translation by the authors; four 7-point items, two from 1 = *extremely undesirable* to 7 = *extremely desirable*, one from 1 = *very much lower than average* to 7 = *very much higher than average*, one from 1 = *very bad catch* to 7 = *very good catch*). Mate value was defined by Fisher et al. (2008, p. 157) as “the total sum of characteristics an individual possesses at a given moment and within a particular context that impacts on their ability to successfully find, attract, and retain a mate.” The Naughty Nine Scale (NNS; Küfner et al., 2015; nine 9-point items from 1 = *does not apply at all* to 9 = *applies completely*) served as a measure of core aspects of the Dark Triad, i.e., narcissism, Machiavellianism, and psychopathy. Finally, the Past Social Deviance Index (PSDI; 20 self-developed 5-point items ranging from 1 = *never* to 5 = *6 times or more*) was used to assess socially undesirable behavior of varying severity in participants’ past. The items were constructed for high face validity (example items: “I have intentionally physically harmed another person [e.g., in a fight, out of anger, to force them to do something, etc.]”; “I have entered or used public transportation, a club, a movie theater, a swimming pool, or similar without paying the required fee”).

#### Manipulation Checks

Following T2, participants answered seven manipulation-check questions, two of which assessed perceived state sexual arousal: “How sexually aroused are you right now?”, i.e., Arousal_Now_, and “How sexually aroused were you directly after hearing the story?”, i.e., Arousal_Story_. Responses were given on a 7-point scale from 1 = *not aroused at all* to 7 = *very aroused*. Three compliance-check questions assessed how attentively participants listened to the audio recording (Q1: “Who seduces whom in the story?”; Q2: “What beverage is being drunk in the story?”; Q3: “Where does the story take place?”). Each question offered four response options (Q1: “A man seduces a woman.”, “A woman seduces a woman.”, “A woman seduces a man.”, “A man seduces a man.”; Q2: “Wine.”, “Milk.”, “Soda.”, “Spirits.”; Q3: “In a cornfield.”, “In an apartment.”, “In a swimming pool.”, “In a disco.”). The questions and response options were intentionally kept simple,^5^ while still requiring participants to have listened to the story until the end. Additionally, participants were explicitly asked, “Were you able to listen to the entire story?” (“yes”/“no”). Finally, the extent to which participants answered truthfully during the study was assessed with the question, “How honestly did you answer the questions throughout this study?” (5-point scale from 1 = *not honest at all* to 5 = *completely honest*).

### Procedure

The study description stated that participants would complete selection tasks, answer questions on sexual attitudes, behaviors, and experiences, respond to items assessing norm violations, and listen to an erotic story. It was specified that the study required downloading the Inquisit 5 Lab software (Millisecond, 2016). The purpose, duration, and content of the download were clearly explained to address potential privacy concerns. Participation using a smartphone or tablet was not permitted. Participants were advised to ensure a quiet and private environment, connect headphones, and adjust the volume to a comfortable level. They were informed that participation was fully anonymous, could be terminated at any time, and that honest responses were essential to ensure the validity of the results, as such instructions have been shown to improve data quality (Vésteinsdóttir et al., 2019). In line with the preregistered sampling plan, an upper limit of 720 participants was set.^6^ The incentive for completing the approximately 30-min study was €5.50 (about US$6 at the time of the study).

Immediately before the study, participants were instructed to download Inquisit 5, which was used to display the instructions, questionnaires, and decision tasks, and to locally record all key presses and RTs on participants’ computers. Participants were then thanked for their interest in the study, and the recruitment information was reiterated in detail. After participants had provided informed consent, sociodemographic data (age, gender, education level, relationship status, and self-assessment on the Kinsey Scale) were collected. Participants then read the description of the upcoming decision task, which instructed them to press one of two keys as quickly as possible to choose between two simultaneously presented images of women, based on the question “Which woman is more likely to flirt with you right now?”

T1 started with four practice trials (unbeknownst to participants and excluded from analysis), followed by 52 regular trials, 26 from the HC condition and 26 from the LC condition, which were presented in random order within the block. After T1, participants completed nine questionnaires (see Measures section for details). Next, they were asked to put on headphones and listen to a 332-s audio narrative of explicit erotic content used to induce sexual arousal (taken from Imhoff & Schmidt, 2014, Study 1). Subsequently, T2 commenced with two practice trials and again 52 regular trials with an identical setup. Finally, participants answered the manipulation-check items (see Measures section for details). The extent of sexual arousal evoked by the erotic audio recording was assessed only after T2 rather than immediately after the audio stimulus, to (1) prevent interruption or distraction between the induction of sexual arousal and the onset of T2 by a question requiring cognitive processing and (2) avoid overly emphasizing the significance of sexual arousal for the subsequent task, thus preempting altered (e.g., inhibited) response behavior.

At the beginning of the key-press task, participants had to press any key, after which the two images of the first practice trial appeared in the upper left and right corners of the screen. They then had to choose one of the two images by pressing a specific key (A for the left image, L for the right image) within a 2500 ms response window. If no image was selected within this window, a prompt in red letters appeared reminding participants to respond faster. Inter-trial intervals randomly varied between 1000 ms, 1500 ms, and 2000 ms to prevent the formation of a fixed response pattern or anticipatory behavior. A shortened version of the instruction (i.e., “Who’s flirting?”) was displayed at the bottom of the task screen during each trial to remind participants of the task at hand.

### Statistical Analyses

#### Dependent Variables

For the key-press task, RT and ER aggregated per trial type and measurement were used as dependent variables. RT (in milliseconds) was defined as the time span between stimulus onset and pressing a key on the participant’s keyboard, indicating a selection of one of the two presented stimuli. An error was defined as a conceptually incorrect choice, i.e., choosing the image with the rejecting facial expression.

For the correlation analyses conducted in this study, the response measures RT and ER were transformed into trial-condition–based difference scores for each measurement (e.g., ΔRT_T1_ = RT_T1_HC_ − RT_T1_LC_). Accordingly, ΔRT and ΔER refer to these simple focal difference scores, representing the selection difficulty arising specifically from the incongruent presentation of global and specific cues; ΔER is taken to indicate individual differences in OGC, whereas ΔRT captures complementary information on conflict-related decision dynamics. To examine conditional effects of sexual arousal within the moderation analyses, a double difference score was used for each response measure, capturing the change in conflict-related selection difficulty over time (e.g., ΔRT_T2–T1_ = ΔRT_T2_ − ΔRT_T1_).

#### Analyses of Variance and Moderation Analyses

To test H1–H3, a 2 (Trial Type: HC vs. LC) × 2 (Time: T1 vs. T2) repeated measures ANOVA was conducted. To examine the presumed moderating effects outlined in H4, we initially preregistered a traditional hierarchical regression approach as used in Landwehr et al. (2024). However, we instead employed Montoya’s (2024) method for moderation analysis in two-instance repeated-measures designs, as it offers several advantages over traditional models. Specifically, this approach is tailored to within-subjects designs with two measurements, avoids inflated error variance that can arise when repeated measures are treated as independent, and provides a direct test of whether individual differences predict change over time. Moreover, the Johnson–Neyman procedure was used to identify the range of the moderator for which the conditional effect of sexual arousal on conflict-related performance was statistically significant, thereby allowing a continuous probing of moderation effects without relying on arbitrary cutoffs. Accordingly, second-order difference scores were regressed on each moderator in separate models to examine moderation effects for RT and ER. To test H6, T2 was split into two halves (T2.1 and T2.2) for each trial type (HC and LC) after 13 trials, marking the midpoint of each trial set. Based on the resulting blocks, a 2 (Trial Type: HC vs. LC) × 2 (Time: T1 vs. T2) × 2 (Block: T2.1 vs. T2.2) repeated measures ANOVA was conducted.

#### Correlation Analyses and General Procedures

To test H5, we originally preregistered a 2 (Time: T1 vs. T2) × 2 (Trial Type: HC vs. LC) × 2 (Conflict Potential: High vs. Low) repeated measures ANOVA. However, we deviated from this plan in favor of a more targeted analysis using Spearman’s rank correlations, which enabled us to examine, for each HC trial, the association between the attractiveness difference of its two images and the corresponding mean RT and ER across participants. A third deviation from the preregistration concerned the operationalization of conflict potential. This adjustment became necessary following the results of the subsequent normative validation study (Landwehr & Landwehr, 2025), which revealed that the Casual × Flirting image was generally perceived as more sexually attractive than its Sexy × Rejecting counterpart.^7^ As a result, the originally intended increase in conflict potential through a stronger sexual distractor was not implemented. Instead, we opted to return to the trial construction logic used in our previous study (Landwehr et al., 2024), whereby conflict intensity within the HC condition was again systematically varied as a function of cue similarity, with trials involving more similar sexual attractiveness ratings for the two images (i.e., smaller attractiveness differences, calculated as Casual × Flirting minus Sexy × Rejecting) being expected to elicit greater selection conflict, resulting in longer RTs and higher ERs.^8^ The sample size per HC image pair (i.e., number of completed trials) ranged from *n* = 265 to 284 but remained consistent for RT and ER, as trials exceeding the 2500 ms limit were coded as missing values for both measures (1.4% of all trials).

As preregistered, for the multiple correlations examined in H7, a correlation coefficient of *r* = .20 was considered the smallest effect size of interest and thus interpretable. Reliabilities (Cronbach’s α) for the difference variables ΔRT_T1_, ΔRT_T2_, ΔER_T1_, and ΔER_T2_ were computed based on four alternating segments of 6 and 7 trials per trial type and response measure. Data from the six practice trials were excluded from all analyses. The significance level for all calculations was set to α = .05. Effect sizes (*r*, *R*^2^, η_p_^2^, *d*_z_, Cohen’s *q*, and β) are reported according to the corresponding statistical method.^9^

## Results

### Mean Differences

First, to test the internal consistency of the paradigm, a 2 (Trial Type: HC vs. LC) × 2 (Time: T1 vs. T2) repeated measures ANOVA was conducted for both RT and ER. As shown in Table 1, a significant main effect of trial type emerged for both response measures. As preregistered, means were higher in the HC condition than in the LC condition, indicating that in the HC condition, participants took longer to make a selection and were more prone to conceptually incorrect choices. Thus, H1 was fully supported. Table 1 also reveals a significant main effect of time for both RT and ER, consistent with H2. As specified in our preregistration, RT decreased from T1 to T2, whereas ER increased.

**Table 1:** Repeated-measures ANOVAs for key-press measures

	RT				ER			
Effect	*F*(1, 283)	*p*		η_p_^2^	*F*(1, 283)	*p*		η_p_^2^
Trial Type	72.94	< .001		.21	53.38	< .001		.16
Time	36.98	< .001		.12	9.55	.002		.03
Trial Type × Time	18.86	< .001		.06	11.25	< .001		.04
Block^†^	0.06	.814		.00	7.73	.006		.03
Trial Type × Block^†^	20.50	< .001		.07	7.39	.007		.03
Time × Block^†^	40.77	< .001		.13	7.22	.008		.03
Trial Type × Time × Block	7.57	.006		.03	8.73	.003		.03
	T1 *M* (*SD*)		T2 *M* (*SD*)		T1 *M* (*SD*)		T2 *M* (*SD*)	
HC	1152 (215)		1103 (249)		.11 (.18)		.14 (.22)	
LC	1137 (202)		1059 (228)		.05 (.11)		.05 (.10)	

*N* = 284. ER = error rate; RT = reaction time; HC = high-conflict trial condition; LC = low-conflict trial condition

Trial Type, Time, and their interaction were estimated using the 2 × 2 GLM model; all other effects were derived from the full 2 × 2 × 2 model including Block

^†^Effects were not part of the preregistered hypotheses and are reported for exploratory purposes

**Fig. 2:**
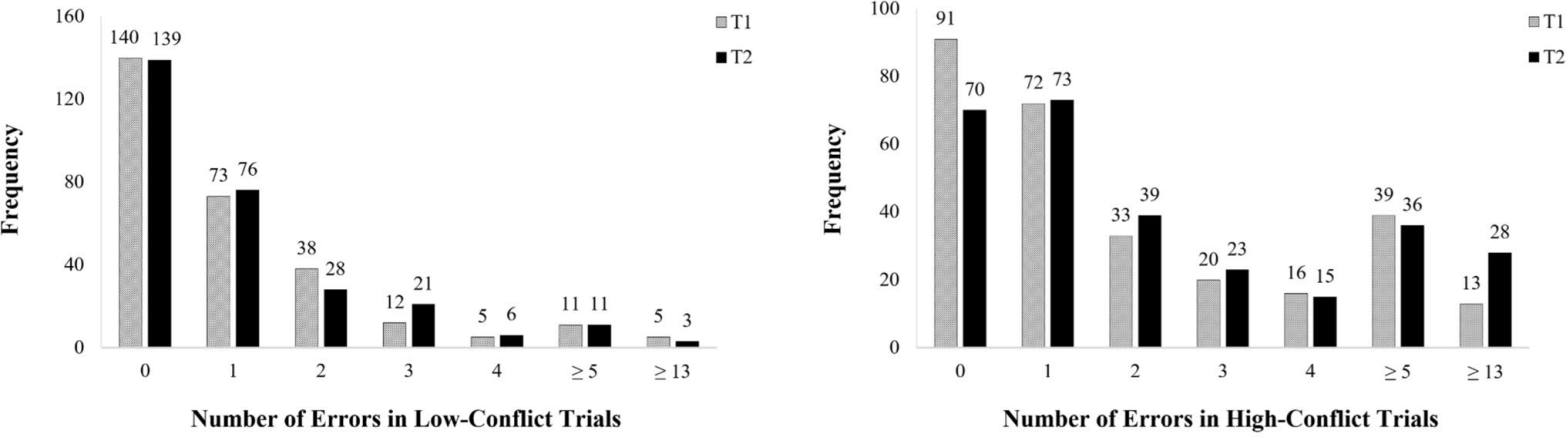
Numbers of errors for both trial conditions

In line with H3, a Trial Type × Time interaction was observed for both RT and ER (Table 1). However, contrary to H3, the decrease in RT was more pronounced for the LC condition (*p* < .001, *d*_z_ = 0.45) than for the HC condition (*p* < .001, *d*_z_ = 0.25). The interaction effect on ER, however, was consistent with our preregistered expectation: In the more conflict-inducing HC condition, ER increased significantly from T1 to T2 (*p* < .001, *d*_z_ = 0.21), whereas in the less demanding LC condition, no statistically significant change occurred (*p* = .711, *d*_z_ = 0.02). Error distributions as a function of trial type and time are presented in Fig. 2.

### Moderation Analyses

**Fig. 3:**
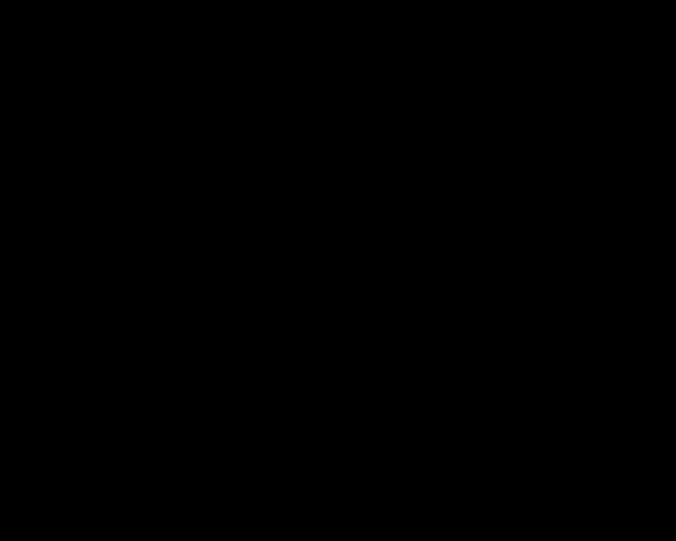
Moderation of the Sexual Entitlement subscale (mean-centered) of the Sexual Narcissism Scale on the relationship between sexual arousal and overreliance on global cues, as indicated by error rates. *Note*. The vertical line marks the Johnson–Neyman criterion, indicating the point on the X-axis beyond which the moderator significantly influences the dependent variable (i.e., where the lower limit of the 95% CI exceeds 0; here: 56% of the sample)

Under H4, we examined various theoretically relevant moderators of the relationship between sexual arousal and OGC. Contrary to our preregistered expectations, most variables did not emerge as significant moderators in the present sample, regardless of whether moderation effects were examined for ER (*p*s ≥ .104) as the primary indicator of OGC or for RT (*p*s ≥ .076) as a complementary measure of decision dynamics. This included sex drive (SDCI), sexual objectification (SOOI), low sexual empathy (SNS_Emp_), Dark Triad traits (NNS), and past socially deviant behavior (PSDI). The only exception was sexual entitlement (SNS_Ent_), for which the Johnson–Neyman technique identified a significance threshold at a (mean-centered) moderator value of − 0.48 (*p* = .030) for OGC as indicated by ER (but not for RT, *p* = .108). Above this threshold, a disposition for sexual entitlement significantly increased the impact of sexual arousal on OGC, with 56% of the sample falling within this range (Fig. 3). Full results of the moderation analyses are presented in Tables 2 and 3.

**Table 2:** Linear regression analyses testing sexual constructs as moderators of the conditional effects of sexual arousal on overreliance on global cues (Montoya, 2024)

	ΔRT_T2–T1_								ΔER_T2–T1_					
Predictor	*R*^2^	ß	*SE*	*t*	*p*	CI_LL_	CI_UL_	*R*^2^	ß	*SE*	*t*	*p*	CI_LL_	CI_UL_
*SDCI*														
Model sum	.00				.677			.01				.162		
Model														
Constant		28.84	6.65	4.34	** < .001**	15.75	41.93		0.04	0.01	3.36	** < .001**	0.02	0.06
SDCI		− 3.48	8.34	− 0.42	.677	− 19.89	12.93		0.02	0.01	1.40	.162	− 0.01	0.05
*SNS*_*Emp*_														
Model sum	.01				.076			.00				.601		
Model														
Constant		28.84	6.62	4.36	** < .001**	15.82	41.86		0.04	0.01	3.35	** < .001**	0.02	0.06
SNS_Emp_		− 19.61	11.01	− 1.78	.076	− 41.28	2.05		0.01	0.02	0.52	.601	− 0.03	0.05
*SNS*_*Ent*_														
Model sum	.01				.108			.02				**.030**		
Model														
Constant		28.84	6.62	4.36	** < .001**	15.81	41.87		0.04	0.01	3.38	** < .001**	0.02	0.06
SNS_Ent_		− 11.71	7.27	− 1.61	.108	− 26.02	2.59		0.03	0.01	2.18	**.030**	0.00	0.05
*SOOI*														
Model sum	.00				.454			.01				.104		
Model														
Constant		28.84	6.65	4.34	** < .001**	15.76	41.92		0.04	0.01	3.36	** < .001**	0.02	0.06
SOOI		6.45	8.59	0.75	.454	− 10.46	23.36		0.02	0.01	1.63	.104	− 0.01	0.05
*SMED*														
Model sum	.00				.744			.02				**.016**		
Model														
Constant		28.84	6.65	4.34	** < .001**	15.75	41.93		0.04	0.01	3.38	** < .001**	0.02	0.06
SMED		− 2.36	7.21	− 0.33	.744	− 16.55	11.84		0.03	0.01	2.42	**.016**	0.01	0.05
*SUSS*^†^														
Model sum	.00				.658			.03				**.002**		
Model														
Constant		28.84	6.65	4.34	** < .001**	15.75	41.93		0.04	0.01	3.41	** < .001**	0.02	0.06
SUSS		− 0.13	0.29	− 0.44	.658	− 0.69	0.44		0.00	0.00	3.13	**.002**	0.00	0.00

*N* = 284. CI_LL_ and CI_UL_ = lower and upper limits of the 95% confidence intervals; ER = error rate; RT = reaction time; SDCI = Sex Drive Composite Index; SMED = Sexual Manipulativeness, Exploitativeness, and Disinhibition Factor; SNS_Emp_ = Sexual Narcissism Scale: Low Sexual Empathy; SNS_Ent_ = Sexual Narcissism Scale: Sexual Entitlement; SOOI = Sexual Objectification of Others Inventory; SUSS = Socially Undesirable Sexual Selection Scale

^†^Predictors were not part of the preregistered hypotheses and are reported for exploratory purposes. Statistically significant results (*p* < .05) are in bold

**Table 3:** Linear regression analyses testing non-sexual constructs as moderators of the conditional effects of sexual arousal on overreliance on global cues (Montoya, 2024)

	ΔRT_T2–T1_								ΔER_T2–T1_					
Predictor	*R*^2^	ß	*SE*	*t*	*p*	CI_LL_	CI_UL_	*R*^2^	ß	*SE*	*t*	*p*	CI_LL_	CI_UL_
*NNS*														
Model sum	.00				.413			.00				.629		
Model														
Constant		28.84	6.64	4.34	** < .001**	15.76	41.92		0.04	0.01	3.35	** < .001**	0.02	0.06
NNS		3.75	4.57	0.82	.413	− 5.25	12.74		0.00	0.01	0.48	.629	− 0.01	0.02
*PSDI*														
Model sum	.00				.313			.00				.956		
Model														
Constant		28.84	6.64	4.34	** < .001**	15.77	41.91		0.04	0.01	3.35	** < .001**	0.02	0.06
PSDI		13.12	12.97	1.01	.313	− 12.40	38.64		0.00	0.02	− 0.06	.956	− 0.04	0.04
*MVS*^†^														
Model sum	.00				.524			.00				.422		
Model														
Constant		12.38	26.67	0.46	.643	− 40.11	64.87		0.00	0.04	0.06	.955	− 0.08	0.09
MVS		3.57	5.60	0.64	.524	− 7.45	14.59		0.01	0.01	0.81	.422	− 0.01	0.03

*N* = 284. CI_LL_ and CI_UL_ = lower and upper limits of the 95% confidence intervals; ER = error rate; RT = reaction time; MVS = Mate Value Scale; NNS = Naughty Nine Scale; PSDI = Past Social Deviance Index

^†^This predictor was not part of the preregistered hypotheses and is reported for exploratory purposes. Statistically significant results (*p* < .05) are in bold

### Impact of Conflict Potential at Trial Level

H5 proposed that higher conflict potential in HC trials—defined as higher cue similarity, i.e., a smaller difference in attractiveness between the two images of a trial, one with revealing clothing and the other with a flirtatious facial expression—would correlate with (1) longer mean RTs and (2) higher mean ERs, both averaged across participants. This preregistered hypothesis was only partially supported by the data. Attractiveness differences showed no meaningful correlation with RTs at T1 (*r* = .08, *p* = .573) and a small negative correlation at T2 (*r* =  − .16, *p* = .257), resulting in an overall near-zero correlation (*r* =  − .06, *p* = .673) across all trials. The difference between the correlations at T1 and T2 was statistically significant (*p* = .005, Cohen’s *q* = 0.24), indicating a notable variation in the relationship between conflict potential and RTs across measurements.

Clearer effects emerged for ERs. At T1, higher cue similarity was associated with moderately higher ERs (*r* =  − .28, *p* = .044), while the corresponding correlation at T2 was weaker (*r* =  − .09, *p* = .526).^10^ The overall correlation across both measurements remained small (*r* =  − .14, *p* = .322). The decline in correlation from T1 to T2 was statistically significant (*p* = .025, Cohen’s *q* = 0.20), suggesting that the impact of conflict potential on decision accuracy diminished over time. In sum, conflict potential within HC trials was related to ERs, but not to RTs, and this relationship was only evident early in the experiment. While the significant Cohen’s *q* values indicate that the strength of the association between cue similarity and the respective response measure changed across measurements, they do not by themselves confirm the presence of any meaningful association.

### Block-Level Analyses Within Measurements

As preregistered, H6 proposed that sexual arousal would be elevated during the initial phase of T2 (T2.1), leading to faster RTs and higher ERs, while a gradual decline in arousal over the course of T2 was assumed to result in slower RTs and lower ERs during its second half (T2.2). This pattern was expected to emerge within both trial types (HC and LC), with within-condition differences anticipated to be more pronounced in HC than in LC trials. The prerequisite for testing H6 was met, as participants reported significantly higher perceived sexual arousal immediately after listening to the erotic story, i.e., just before T2 (Arousal_Story_; *M* = 4.27, *SD* = 2.00), than directly after T2 (Arousal_Now_; *M* = 3.56, *SD* = 1.81), *p* < .001, *d*_z_ = 0.75. Taken together, the higher arousal reported as a result of the audio stimulus and the significant decline across T2 indicate that arousal was indeed elevated at the onset of T2, as otherwise the observed decrease would imply values falling below baseline by the end of T2, which is implausible.

#### Reaction Time Differences Across Blocks

Beyond the main effects and the Trial Type × Time interaction reported above (see Table 1), the current analysis focused on changes in RTs between the first (T2.1) and second (T2.2) half of T2.^11^ A 2 (Trial Type: HC vs. LC) × 2 (Time: T1 vs. T2) × 2 (Block: T2.1 vs. T2.2) repeated measures ANOVA revealed a significant three-way interaction, *F*(1, 283) = 7.57, *p* = .006, η_p_^2^ = .03. To disentangle this effect, estimated marginal means were analyzed separately for HC and LC trials. Bonferroni-adjusted pairwise comparisons were used to examine changes across adjacent measurement blocks within each trial type. In LC trials, RT decreased significantly from T1.1 to T1.2 (Δ*M* =  − 42.27 ms, *p* < .001, *d*_z_ = 0.28) and again from T1.2 to T2.1 (Δ*M* =  − 63.52 ms, *p* < .001, *d*_z_ = 0.33), but then remained stable from T2.1 to T2.2 (Δ*M* =  + 13.53 ms, *p* = .571, *d*_z_ = 0.10). In HC trials, RT showed a comparable decrease from T1.1 to T1.2 (Δ*M* =  − 32.50 ms, *p* = .002, *d*_z_ = 0.19) and from T1.2 to T2.1 (Δ*M* =  − 60.64 ms, *p* < .001, *d*_z_ = 0.30), but unlike in LC trials, RTs increased from T2.1 to T2.2 (Δ*M* =  + 55.79 ms, *p* < .001, *d*_z_ = 0.37).

**Fig. 4:**
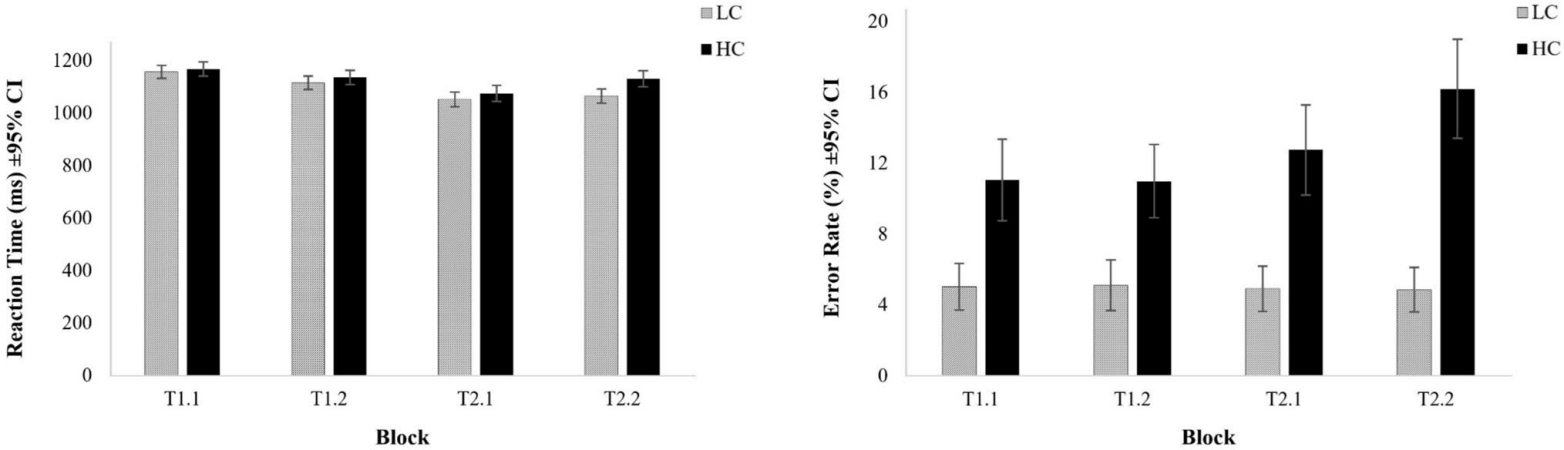
Response measures across blocks

This divergence resulted in differentiated trajectories: While the RT decrease in LC trials observed until T2.1 leveled off between T2.1 and T2.2, HC trials exhibited a decline until T2.1 followed by an increase from T2.1 to T2.2 (see Fig. 4). Although the latter pattern is descriptively consistent with the assumption of a diminishing influence of sexual arousal over time, the results do not support H6 as preregistered, as the Trial Type × Time interaction was not significantly more pronounced in the first half of T2 than in the second half.

#### Error Rate Differences Across Blocks

A parallel analysis was conducted for ERs to test whether the pattern observed for RTs also applied to accuracy. The repeated measures ANOVA revealed a significant Trial Type × Time × Block interaction, *F*(1, 283) = 8.73, *p* = .003, η_p_^2^ = .03. Bonferroni-adjusted pairwise comparisons were then conducted within each trial type to further examine this effect. In LC trials, ER remained stable from T1.1 to T1.2 (Δ*M* =  + 0.09%, *p* = .845, *d*_z_ =  − 0.01), from T1.2 to T2.1 (Δ*M* =  − 0.19%, *p* = .726, *d*_z_ = 0.02), and from T2.1 to T2.2 (Δ*M* =  − 0.06%, *p* = .902, *d*_z_ =  − 0.01).^12^ ER in HC trials remained stable from T1.1 to T1.2 (Δ*M* =  − 0.06%, *p* = .935, *d*_z_ = 0.01) and from T1.2 to T2.1 (Δ*M* =  + 0.18%, *p* = .067, *d*_z_ =  − 0.11), but, in contrast to RT, showed a significant increase from T2.1 to T2.2 (Δ*M* =  + 3.45%, *p* = .003, *d*_z_ =  − 0.30).

Together, these findings reveal a distinct developmental pattern: In LC trials, ER remained stable across all blocks, whereas in HC trials, ER increased markedly in the second half of T2. Contrary to H6, this pattern does not indicate a diminishing influence of sexual arousal over time. The interaction is illustrated in Fig. 4. The exploratory results of the main and interaction effects involving Block (i.e., Block, Trial Type × Block, and Time × Block) are summarized in Table 1.

### Nomological Network of Overreliance on Global Cues and Problematic Sexuality

One of the aims of the current study was to examine whether OGC, as assessed by key-press measures, can be meaningfully embedded within a broader nomological network of problematic sexuality, as reflected in established self-report constructs (H7). To this end, we computed correlations between the key-press difference scores (ΔRT and ΔER) and a range of self-report measures (Table 4). The presence of systematic and theoretically plausible associations would support the assumption that OGC, as an experimental construct, is aligned with established dispositional indicators of interpersonally problematic sexual behavior. At the same time, such a pattern would also provide evidence for the convergent validity of the current paradigm, indicating that the behavioral indices meaningfully converge with self-reported traits.

**Table 4:** Correlations between self-report measures and key-press difference scores by measurement

	T1		T2	
	ΔRT	ΔER	ΔRT	ΔER
MVS	.04	− .07	.08	− .01
NNS	− .06	− .06	.01	.07
PSDI	− .08	.01	.01	.01
SDCI	.07	.19**	.03	.22***
SDI-2	.02	.18**	.03	.16**
SES	.03	.15*	.02	.21***
SMED^†^	.07	.21***	.04	.28***
SNS_Emp_	.11	.11	− .04	.12*
SNS_Ent_	.12*	.24***	− .02	.30***
SOI-R	.12*	.11	.03	.14*
SOOI	.01	.16**	.07	.21***
SUSS	.05	.14*	.01	.26***
Arousal_Story_	− .03	.11	.02	.23***
Arousal_Now_	.00	.10	.01	.28***
Age	.10	− .03	.01	.00
EA	.03	− .18**	.13*	− .05
RS	.14*	− .07	.07	.04
Reliability (α)	.29	.85	.30	.91

*N* = 284. ER = error rate; RT = reaction time; EA = educational attainment; RS = relationship status; MVS = Mate Value Scale; NNS = Naughty Nine Scale; PSDI = Past Social Deviance Index; SDCI = Sex Drive Composite Index; SDI-2 = Sexual Desire Inventory-2; SES = Sexual Excitation Scale; SMED = Sexual Manipulativeness, Exploitativeness, and Disinhibition Factor; SNS_Emp_ = Sexual Narcissism Scale: Low Sexual Empathy; SNS_Ent_ = Sexual Narcissism Scale: Sexual Entitlement; SOI-R = Revised Sociosexual Orientation Inventory; SOOI = Sexual Objectification of Others Inventory; SUSS = Socially Undesirable Sexual Selection Scale

^†^The SMED Factor was not part of the preregistered hypotheses and is reported for exploratory purposes. Statistically significant results (*p* < .05) are in bold

^*^p < .05; ** *p* < .01; *** *p* < .001

A clear pattern of positive correlations emerged between ΔER and those self-report measures assessing sexual individual differences (i.e., SDCI, SDI-2, SES, SNS_Emp_, SNS_Ent_, SOI-R, SOOI, and SUSS), as well as the two measures of sexual arousal (Arousal_Story_ and Arousal_Now_), with effect sizes ranging from *r* = .10 to .30 (Table 4). This distinct pattern of convergent validity was evident for ΔER at both T1 and T2, though more pronounced at T2. At T2, all possible correlations between ΔER and the sexual self-report and arousal measures were significantly positive, with seven exceeding .20, whereas at T1, only 60% of the correlations were significant, with just one above .20. In contrast to the sexual measures, no meaningful correlations were observed between ΔER and the nonsexual individual differences (MVS, NNS, PSDI) or the demographic variables (age, educational attainment, and relationship status). Unlike ΔER, ΔRT did not yield any meaningful correlation patterns, showing no significant associations with any individual differences examined. Reflecting the patterns of convergent validity, the internal consistencies of ΔER were good at T1 (α = .85) and excellent at T2 (α = .91), whereas ΔRT was found to be thoroughly unreliable (Table 4).

### Intercorrelations Among Key-Press Measures and Self-Report Measures

Table 5 shows significant positive correlations between the scores of the same response measure at T1 and T2 within the same trial condition (e.g., between ER_LC_T1_ and ER_LC_T2_), confirming the temporal stability of these indices (i.e., rank-order stability). Furthermore, Table 5 indicates that no meaningful intercorrelations (− .08 ≤ *r*s ≤ − .02) exist between RT and ER, regardless of trial type or measurement. The intercorrelations among the measured scales (MVS, NNS, PSDI, SDI-2, SES, SNS_Emp_, SNS_Ent_, SOI-R, SOOI, and SUSS), along with their means, standard deviations, and internal consistencies (αs ≥ .71), are presented in Table 6. As anticipated, the scales assessing sexual individual differences were strongly intercorrelated, providing post hoc support for the formation of an aggregated sex drive index (SDCI; composed of SDI-2, SES, and SOI-R) to be used as a moderator. The NNS, which captures aspects of the Dark Triad, showed significant positive correlations with all scales assessing sexual constructs, the MVS, the PSDI, and both measures of sexual arousal (Arousal_Story_ and Arousal_Now_), while also being significantly negatively correlated with participant age. In contrast, the MVS did not display a consistent correlation pattern and showed only sporadic associations with the other scales. A meaningful correlation of the MVS was found solely with participants’ relationship status, which is conceptually plausible, as individuals with higher MVS scores are more likely to be in relationships—either because high mate value facilitates relationship formation (Apostolou & Michaelidou, 2024) and mate retention (Miner et al., 2009) or because being in a relationship enhances perceived mate value (Yorzinski & Platt, 2010).

**Table 5:** Intercorrelations of key-press measures within the same trial type

	RT_T1_		ER_T1_		RT_T2_		
	HC	LC	HC	LC	HC	LC	
ER_T1_	− .02	− .02					
RT_T2_	.66***	.68***	− .04	− .08			
ER_T2_	− .02	− .02	.70***	.71***	− .13*	− .07	

*N* = 284. ER = error rate; RT = reaction time; HC = high-conflict trial condition; LC = low-conflict trial condition. Statistically significant results (*p* < .05) are in bold

^*^p < .05; ** *p* < .01; *** *p* < .001

**Table 6:** Intercorrelations of self-report measures

	*Min*	*Max*	*M*	*SD*	MVS	NNS	PSDI	SDCI	SDI-2	SES	SMED	SNS_Emp_	SNS_Ent_	SOI-R	SOOI	SUSS	Arousal_S_	Arousal_N_	Age	EA
MVS	1.00	7.00	4.61	1.19	(.86)															
NNS	1.00	8.44	3.94	1.46	.17**	(.83)														
PSDI	1.00	3.50	1.67	0.51	.04	.36***	(.83)													
SDCI	− 1.92	2.23	0.00	0.80	.12*	.32***	.24***	(.71)												
SDI-2	1.63	7.88	5.05	1.17	.15*	.26***	.11	.84***	(.79)											
SES	1.00	5.00	3.10	0.82	.02	.24***	.14*	.81***	.58***	(.80)										
SMED^†^	− 2.13	2.84	0.00	0.92	.07	.45***	.27***	.86***	.68***	.71***	–									
SNS_Emp_	1.00	3.80	1.77	0.60	− .20***	.21***	.02	.20***	.06	.20***	.38***	(.73)								
SNS_Ent_	1.00	5.00	1.90	0.91	.09	.31***	.04	.37***	.24***	.35***	.59***	.37***	(.85)							
SOI-R	1.22	5.00	3.06	0.79	.11	.28***	.33***	.75***	.43***	.36***	.67***	.22***	.30***	(.81)						
SOOI	1.20	5.00	2.88	0.77	.04	.40***	.26***	.63***	.54***	.47***	.87***	.31***	.44***	.50***	(.82)					
SUSS	0.00	100.00	40.88	23.32	.05	.43***	.25***	.59***	.42***	.50***	.83***	.28***	.46***	.50***	.62***	(.95)				
Arousal_S_	1.00	7.00	4.27	2.00	.13*	.12*	.04	.40***	.35***	.44***	.37***	.06	.15**	.18**	.25***	.30***	–			
Arousal_N_	1.00	7.00	3.56	1.81	.12*	.16**	.05	.46***	.41***	.49***	.43***	.16**	.19**	.22***	.29***	.33***	.87***	–		
Age	18.00	73.00	29.86	9.04	− .07	− .24***	.10	.10	.07	.01	.10	− .02	− .03	.16**	.15*	.07	.09	.07	–	
EA	1.00	6.00	5.35	0.80	.19**	.01	− .01	.11	.12	.00	.08	− .06	− .05	.15*	.10	.04	− .05	− .03	.07	–
RS	1.00	2.00	1.61	0.49	.39***	.06	− .01	.11	.19**	.01	.08	− .17**	.07	.08	.03	.09	.04	.03	.16**	.32***

*N* = 284. Arousal_S_ = Arousal_Story_; Arousal_Now_ = Arousal_N_

EA = educational attainment; RS = relationship status; MVS = Mate Value Scale; NNS = Naughty Nine Scale; PSDI = Past Social Deviance Index; SDCI = Sex Drive Composite Index; SDI-2 = Sexual Desire Inventory-2; SES = Sexual Excitation Scale; SMED = Sexual Manipulativeness, Exploitativeness, and Disinhibition Factor; SNS_Emp_ = Sexual Narcissism Scale: Low Sexual Empathy; SNS_Ent_ = Sexual Narcissism Scale: Sexual Entitlement; SOI-R = Revised Sociosexual Orientation Inventory; SOOI = Sexual Objectification of Others Inventory; SUSS = Socially Undesirable Sexual Selection Scale

^†^The SMED Factor was not part of the preregistered hypotheses and is reported for exploratory purposes. Statistically significant results (*p* < .05) are in bold. Cronbach’s α is given in brackets

*p < .05; ** *p* < .01; *** *p* < .001

The two measures of sexual arousal, Arousal_Story_ and Arousal_Now_, exhibited a positive correlation pattern with most scales assessing sexual constructs (Table 6), indicating a plausible link between dispositional sexual arousability and individual differences in problematic sexuality. Additionally, the two arousal measures correlated with the two nonsexual scales, MVS and NNS, and showed a strong intercorrelation. Regarding the biographical variables, age, educational attainment, and relationship status showed no notable correlations with the assessed scales, the arousal measures, or each other, except for the associations between age and NNS, as well as relationship status and MVS (Table 6).

### Non-Preregistered Exploratory Analyses

Given the substantial intercorrelations among the seven self-report measures assessing problematic sexuality in the present study (i.e., SDI-2, SES, SNS_Emp_, SNS_Ent_, SOI-R, SOOI, and SUSS), an exploratory principal axis factor analysis was conducted to explore their underlying structure and determine whether a latent factor could capture shared variance across these constructs. The KMO measure of sampling adequacy was .823, indicating that the data were well suited for factor analysis. Bartlett’s test of sphericity was significant, χ^2^(21) = 675.05, *p* < .001, indicating that the inter-item correlations were sufficiently large. The communalities for six of the seven measures fell within a moderate to good range (.305 to .542), except for SNS_Emp_, which showed a substantially lower communality of .184, suggesting a more distinct construct compared to the others. The initial eigenvalues pointed to one dominant factor, accounting for 49% of the variance, with a second factor explaining an additional 16%. However, the scree plot revealed a clear elbow at the first factor, thus supporting the decision to retain a one-factor solution. The extracted factor loaded strongly on measures reflecting manipulative, exploitative, and disinhibited sexual tendencies and was thus labeled Sexual Manipulativeness, Exploitativeness, and Disinhibition (SMED).

The SMED factor was used in subsequent analyses to examine its correlations with key-press difference scores (Table 4) and with individual self-report measures (Table 6). Regarding the key-press difference scores, a pattern consistent with the correlations between individual self-report measures and key-press difference scores at T1 and T2 (Table 4) emerged, once again underscoring the diagnostic significance of ER difference scores as indicators of OGC, as conceptualized in the present study. In addition to the expected correlations with individual self-report measures of problematic sexuality, the SMED factor also showed significant correlations with the nonsexual measures NNS (*r* = .45) and PSDI (*r* = .27).

Moreover, the SMED factor, the SUSS, and the MVS were exploratively examined as potential moderators of the relationship between sexual arousal and OGC. For the SUSS, the Johnson–Neyman technique identified a significance threshold at − 9.52 (*p* = .002) for OGC as indicated by ER, with 64% of participants exceeding this threshold (Fig. ES1), but not for RT (*p* = .658). Similarly, for the SMED factor, a threshold at − 0.45 (*p* = .016) was found for ER, with 68% of participants within the significant range (Fig. ES2), but again not for RT (*p* = .744). In contrast, no significant moderation effects were found for self-reported mate value, neither for ER (*p* = .422) nor for RT (*p* = .524). In sum, these findings are consistent with the preregistered assumption that individuals with higher levels of sexually manipulative or exploitative tendencies are more susceptible to increased OGC under sexual arousal. Full statistical results of the exploratory moderation analyses are reported in Tables 2 and 3.

## Discussion

### Confirming the Effect of Cue Incongruence via the Key-Press Approach

Our first hypothesis, as preregistered, proposed that RT and ER would differ between the two trial conditions, HC and LC. Since we assumed that incongruent HC trials would be more difficult to resolve than congruent LC trials—due to the sexual distractor competing with the conceptually correct choice—we expected both RT and ER to be higher in the HC condition. Consistent with our predictions and earlier findings (Landwehr et al., 2024; Smith et al., 2018), congruency effects were found for both response measures, as RT and ER were indeed higher in the HC condition, i.e., participants took longer to make a choice and made more errors than in the LC condition. This finding also aligns with recent evidence showing that sexual stimuli reliably prolong decision times in male (and female) participants, underlining their potency as attentional distractors (Imhoff et al., 2020). In light of these results, the simplified key-press approach appears well suited to capturing OGC, with the two response measures providing complementary information about misclassification (ER) and decision difficulty (RT).

The significant main effect of trial type is noteworthy given that each trial featured the same woman in both images, who had been instructed to convey two diametrically opposed affective expressions (sexual interest in one image and firm rejection in the other). One might have reasonably expected that such a clear juxtaposition of two images, whose differences are based solely on two cues (i.e., the global clothing cue and the specific facial expression cue), would facilitate the selection of the conceptually correct option and, in particular, would not produce any systematic errors in the behavioral outcome, regardless of trial type. However, as previously demonstrated (Landwehr et al., 2024) and corroborated by the current findings, even clear affective cues displayed by a woman do not reliably lead male participants to make the stereotypically correct choice (i.e., the choice that aligns with the given instruction and is socially desirable) when these informative affective cues compete with simultaneously occurring non-informative yet sexually appealing ones.

In this context, it is also worth noting that the present findings pertain to conditions of cue incongruence, rendering OGC empirically observable in HC trials, primarily via ERs. Importantly, this does not imply that OGC is absent in cue-congruent situations as reflected by LC trials; rather, its effects are less readily detectable when global and specific cues converge, since in such cases, no OGC-related errors can manifest at the outcome level. In everyday interactions that are characterized by cue congruence (e.g., flirtatious affect accompanied by sexually suggestive attire), differences in cue weighting may thus remain opaque to interaction partners, as approach behavior could be guided predominantly by global sexual cues without directly contradicting affective signals. While such situations do not give rise to overt misclassification, they may nevertheless mask underlying deficits in sensitivity to interaction-specific cues and thereby delay the emergence of detrimental social consequences—for instance, if relationship expectations regarding perspective-taking, responsiveness, or mutual attunement are not met.

### Sexual Arousal Amplifies Overreliance on Global Cues

With our second hypothesis, we addressed the presumed modulatory effect of sexual arousal on OGC. As in our previous study (Landwehr et al., 2024), we assumed that during sexual arousal, changes in the response measures would occur due to motivational states influencing decision-making and selection processes toward need-congruent stimuli (e.g., Seibt et al., 2007). The results show that, consistent with our preregistered predictions, sexual arousal led to a decrease in RT as well as an increase in ER from T1 to T2. Thus, when sexually aroused, participants made their decisions more quickly, and these decisions were more likely to be conceptually incorrect, as they violated the given instruction and thereby contravened the implicitly embedded social norm of considering another person’s intentions. The increase in ER challenges the interpretation of the decreased RT as a sign of performance improvement due to a practice effect and instead suggests that the likelihood of OGC increases as a result of more disinhibited response behavior under sexual arousal (Imhoff & Schmidt, 2014; Wiemer et al., 2023). This interpretation aligns with the results of Bouffard and Miller (2014), who reported that sexual arousal in men is associated with an overperception of women’s sexual intent.

### Interaction Between Cue Combination and Sexual Arousal: Disruptions in Cue-Based Decision-Making

Our third hypothesis posited an interaction between trial type and time, such that the effect of sexual arousal would be more pronounced in HC trials than in LC trials. As preregistered, sexual arousal led to a greater increase in ER in the HC condition than in the LC condition. As also expected, RT decreased from T1 to T2 in both trial conditions; however, contrary to our hypothesis, this decrease was more pronounced in the LC condition than in the HC condition. One possible explanation is that the elevated decisional conflict inherent in HC trials may have constrained the magnitude of arousal-related response acceleration. In such trials, in which response selection requires resolving competing cues, the facilitating effect of sexual arousal on response speed may have been partially offset by increased processing demands, resulting in a comparatively smaller RT decrease. Despite this unexpected result, the findings support the broader conclusion that sexual arousal impairs socially appropriate decision-making under cue incongruence while generally accelerating selection behavior.

Complementary theoretical perspectives help contextualize these effects. Research on motivated social categorization suggests that individuals tend to categorize others based on goal-relevant characteristics linked to perceived desirability (Maner et al., 2005, 2012). During sexual arousal, sexually salient cues gain increased hedonic significance, as their gratification potential becomes more immediately apparent (Loewenstein, 1996). In this state, projecting one’s own desires onto a sexually desired person may promote the subjective perception of increased sexual availability, even when the displayed affect does not objectively support such an inference (i.e., confirmation bias; Oeberst & Imhoff, 2023). Cues that contradict one’s own goals may therefore receive less attention or be assigned less decisional weight, which provides a plausible account for the observed increase in ER during sexual arousal. While such biases may be evolutionarily adaptive in mate selection (e.g., Haselton & Buss, 2000), they also increase the risk of sexually problematic behavior toward a woman who is in fact sexually disinterested, particularly given the heightened likelihood of male frustration following later rejection (Treat et al., 2020). This interpretation is consistent with Toates’ (2009) hierarchical model of sexual motivation, which posits that incentive-based cues acquire heightened influence when they align with pre-existing motivational states. Within this framework, sexual arousal is expected to enhance OGC, as the motivational salience of global sexual cues increases while more diagnostic affective cues are deprioritized. Together, these accounts highlight how arousal-driven top-down processes can override bottom-up information, reinforcing state-congruent expectations and increasing susceptibility to OGC in sexually charged contexts.

As in the previous study (Landwehr et al., 2024), it constitutes a methodological advantage that differences between trial types and over time (i.e., between visceral states) are captured by the error measure, a behavioral indicator that is unlikely to be influenced by social desirability bias. Although sexual arousal was associated with a substantial relative increase in ER, the low absolute ERs indicate that the task was generally comprehensible and manageable for nonclinical participants. At the same time, the distribution of errors in the HC condition at T2 revealed a subgroup of participants exhibiting notably elevated ERs (i.e., ≥ 13 errors, corresponding to at least 50% of HC trials). This subgroup comprised 9.9% of participants at T2, compared to 4.6% at T1. A comparable subgroup had already emerged in the previous study (Landwehr et al., 2024). Replicating this subgroup effect supports the notion that, for some men, sexual arousal substantially amplifies state-congruent top-down expectations, thereby overriding more informative bottom-up cues (Freeman & Johnson, 2016). It is evident that such a mechanism of (impulsive) sexual selection, when already observable under controlled experimental conditions, may translate into increased individual susceptibility to OGC—and its potential negative consequences—in real-life social situations.

### Identifying Moderators of Sexual Arousal Effects on Overreliance on Global Cues

Under H4, we proposed several potential moderators of the relationship between sexual arousal and OGC. However, the moderation effects observed in the previous study (Landwehr et al., 2024) regarding sex drive (assessed via an aggregate index comprising SDI-2, SES, and SOI-R) and sexual objectification (assessed by the SOOI) were not replicated in the current, higher-powered sample. Likewise, no moderation effects emerged for the nonsexual constructs of antagonistic personality traits (i.e., aspects of the Dark Triad; assessed by the NNS) and proclivity for norm violations (assessed by the PSDI). Furthermore, sexual narcissism did not moderate the effect of sexual arousal on OGC. However, a significant moderating effect was observed for the SNS subscale Sexual Entitlement, which assesses the belief that the fulfillment of one’s sexual desires is a personal right (e.g., ‘‘I am entitled to sex on a regular basis’’; Widman & McNulty, 2010).

Building on this finding, we explored whether the SUSS would also act as a moderator, as it captures manipulative flirting behavior and is thus conceptually adjacent to the propensity for sexual entitlement. Indeed, a pronounced moderation effect of the SUSS on the relationship of interest was found. The resulting assumption that manipulative and exploitative sexual attitudes, motivations, and behaviors—amplified by individual sexual disinhibition—may constitute a central moderating variable was further substantiated by an exploratory analysis examining the SMED factor (see Exploratory Analyses section) as a potential moderator, revealing a significant moderation effect on the relationship between sexual arousal and OGC. The extracted SMED factor thus represents a conceptually coherent aggregate that may facilitate future research on OGC and problematic sexuality while reducing the need for multiple comparisons.

### A Closer Look at Conflict Potential and the Dynamics of Sexual Arousal

The findings for H1 regarding the general effects of (in)congruent cue combinations were complemented by the trial-level results of H5. In these analyses, conflict potential within HC trials was operationalized as cue similarity, i.e., the difference in (average prerated) sexual attractiveness between the conceptually correct (Casual × Flirting) and the incorrect (Sexy × Rejecting) image. Smaller difference scores thus indicated higher cue similarity and, by extension, greater selection conflict. Accordingly, we expected higher cue similarity to be associated with longer RTs and higher ERs.

Contrary to expectations, cue similarity showed no meaningful relationship with RT. Correlations were close to zero at T1 and remained small at T2, indicating that the degree of conflict potential had little to no influence on response speed. In contrast, the results for ER revealed a more differentiated pattern. Higher cue similarity was significantly associated with increased ERs at T1, although this relationship was no longer evident at T2. This attenuation suggests that sexual arousal may have disrupted participants’ sensitivity to cue similarity, weakening the impact of trial-level conflict potential on task accuracy. When sexual arousal increases, participants appear to become less sensitive to selection conflict arising from high cue similarity and generally more susceptible to cue incongruence, even in trials in which normative cue clarity would, under baseline conditions, promote correct responses.

H2 was expanded by introducing an interaction hypothesis (H6) addressing how the influence of sexual arousal on OGC would evolve over the course of T2 across HC and LC trials. Specifically, we hypothesized that the effect of sexual arousal would be strongest immediately after the erotic story and would gradually decline during T2. Participants were therefore expected to be more susceptible to OGC—i.e., to make faster decisions and more errors—during the first half of T2 (T2.1) than during the second half (T2.2), particularly in HC trials. Additional rationale for this hypothesis was provided by prior work on habituation effects following repeated exposure to erotic stimuli (e.g., Dawson et al., 2013; O’Donohue & Geer, 1985).

While the assumption of decreasing sexual arousal during T2 was supported by the manipulation-check measures (Arousal_Story_, Arousal_Now_), the ANOVA results did not support H6. In LC trials, RT declined from T1.2 to T2.1 and then leveled off between T2.1 and T2.2. In HC trials, RT declined from T1.2 to T2.1 but then increased from T2.1 to T2.2, resulting in an increasing divergence between HC and LC trials. The findings for ER deviated even more clearly from the hypothesized pattern, as ER did not decline over time in either condition. In LC trials, ER remained stable across all blocks, i.e., from T1.1 to T2.2. In HC trials, by contrast, ER showed a slight increase from T1.2 to T2.1 and then rose significantly from T2.1 to T2.2. Together, these results contradict the central claim of H6 and instead suggest that the difference in ER between trial types became more pronounced toward the end of the experiment.

One explanation for this pattern is that, while sexual arousal declined during T2, other factors such as fatigue, boredom, or impatience gradually gained influence, particularly in the more demanding HC condition. The concurrent increase in both RT and ER in later HC trials may reflect reduced processing efficiency or diminished task engagement rather than arousal-induced impulsivity. Instead of indicating faster responding at the expense of accuracy, this pattern suggests a late-stage performance decline under sustained high-conflict demands, resulting in slower and more error-prone responses. Research on repetitive categorization tasks supports this interpretation, showing that such tasks can induce boredom and disengagement (Meier et al., 2024). This view is also consistent with findings indicating that habituation to sexual stimuli may involve a shift in attentional focus from a participant perspective to a spectator perspective (Dekker & Everaerd, 1988; Koukounas & Over, 1993).

An alternative possibility is that sexual arousal remained relatively stable throughout T2 but was gradually modulated by additional factors that impaired decision accuracy. In this scenario, sexual arousal may have continued to influence response tendencies, while fatigue or impatience further increased susceptibility to errors. Although the manipulation-check measures suggest a decline in sexual arousal over T2, physiological arousal may have remained elevated, reflecting a potential dissociation between subjective experience and physiological activation (e.g., Chivers et al., 2010). However, this interpretation remains speculative, as physiological arousal was not assessed in the present study.

Regardless of the exact mechanisms underlying the response pattern during T2, the positive correlations between ER at T2 and individual differences linked to problematic sexuality (Table 4) indicate that the observed errors do not reflect random responding. Accordingly, the present paradigm does not merely capture nonspecific performance deterioration driven by impatience or boredom (Moynihan et al., 2017). Rather, the results suggest that men’s susceptibility to OGC under sexual arousal is systematically shaped by stable sexual dispositions.

### Correlational Evidence Linking Overreliance on Global Cues to Problematic Sexuality

In H7, we hypothesized a meaningful pattern of positive correlations between the two key-press measures, RT and ER, and a set of self-report measures assessing potentially problematic sexual attitudes, behaviors, and motivations (i.e., SDCI, SDI-2, SES, SNS_Emp_, SNS_Ent_, SOI-R, SOOI, and SUSS), as well as the two self-report state measures of sexual arousal (Arousal_Story_, Arousal_Now_). Such a pattern had been clearly demonstrated for ΔER in the previous study (Landwehr et al., 2024). Additionally, we assumed conceptual overlaps with (1) aspects of the Dark Triad (i.e., NNS) and (2) past socially deviant behavior (i.e., PSDI), as both are characterized by boundary violations, a more egocentric, utility-oriented perspective, and exploitative tendencies. We also considered a potential association with mate value (i.e., MVS), consistent with findings linking men’s self-perceived mate value to hostile sexism and misogynistic attitudes toward women (Bosson et al., 2022).

The analysis revealed a clear difference in the correlation patterns of the two key-press measures. Consistent with earlier findings (Landwehr et al., 2024), ΔRT proved unreliable and showed no significant associations with sexual or nonsexual measures, nor with the biographical variables. This unreliability aligns with known limitations of difference scores (Thomas & Zumbo, 2012), particularly concerning RT (Draheim et al., 2019). In addition, RT showed no meaningful intercorrelations with ER, regardless of trial type or measurement. This pattern suggests minimal overlap in the explanatory value of the two response measures and provides no evidence for a pronounced speed-accuracy trade-off. Notably, the relationship between RT and ER is not necessarily linear and may therefore not be immediately apparent (Draheim et al., 2019).

In contrast to ΔRT, ΔER showed a robust pattern of associations with measures of problematic sexuality and sexual arousal, with correlations generally stronger at T2 than at T1. Moreover, ΔER demonstrated good (at T1) and excellent (at T2) internal consistency. These findings provide further evidence for the convergent validity of the present paradigm. No substantial correlations emerged between ΔER and nonsexual self-report measures, mate value, or biographical variables, supporting discriminant validity and indicating that OGC reflects interindividual differences specifically related to problematic sexuality rather than general socially undesirable traits. This interpretation aligns with prior work showing that men who engage in sexually coercive behavior (Farris et al., 2008) or endorse rape-supportive attitudes (Treat et al., 2017) rely less on women’s affective cues and place greater emphasis on non-affective cues when evaluating women’s sexual interest.

### Limitations

As with most experimental studies, certain limitations must be considered when interpreting the present findings. First, we relied on an online sample to examine the effects and relationships relevant to our research questions. Although online surveys have traditionally been criticized for potential undercoverage and self-selection bias (Bethlehem, 2010), undercoverage has become negligible in many countries due to the widespread use of the internet (e.g., only around 5% of individuals aged 16 to 74 in Germany had never used the internet in 2023; Statistisches Bundesamt, 2024). Moreover, commercial online panels have been shown to exhibit psychometric properties and criterion validities comparable to conventionally recruited samples (Walter et al., 2019). In line with this, Prolific has repeatedly been found to provide high data quality across key indicators such as attention, comprehension, honesty, and reliability (Peer et al., 2021).^13^

A key advantage of online data collection is the efficient recruitment of large, pre-screened samples. Accordingly, the present study achieved a substantially larger sample size than the previous study (Landwehr et al., 2024), increasing the number of participants by approximately 260% (from *N* = 79 to 284). Combined with the within-subjects design—known to provide higher statistical power than between-subjects designs by reducing between-participant variance (Maxwell & Delaney, 2004)—this renders the present findings more robust than those of the earlier study.

Another limitation concerns the fixed order of arousal states, with sexual arousal solely induced after the baseline block. Although T1 served as a within-participant control condition, this design precludes a complete separation of arousal effects from potential order-related influences. However, the selective performance changes observed at T2 in HC but not LC trials argue against nonspecific disengagement effects (e.g., fatigue- or boredom-related), which would be expected to result in generalized increases in ER across trial types, and against learning effects, which would be expected to manifest as uniform performance improvements. Future studies could further address this issue by counterbalancing the order of arousal induction or including an additional control condition at T2.

Finally, the self-selection inherent in online recruitment resulted in a convenience sample that meets WEIRD criteria (Henrich et al., 2010), with a relatively high proportion of academics. While this limits generalizability (Hanel & Vione, 2016), it is noteworthy that both the core assumptions of the paradigm (H1–H3) and the correlational relationships with problematic sexuality (H7) were replicated in this comparatively homogeneous sample. Importantly, the collected data showed sufficient variance to reveal interindividual differences in susceptibility to OGC under sexual arousal. Concerns that sexual arousal induced “at a distance” might be insufficient were not supported, as indicated by the manipulation checks (Arousal_Story_ and Arousal_Now_) and the observed effects on the dependent measures. Indeed, inducing sexual arousal via erotic media, already well established in laboratory settings (Both et al., 2004), may be particularly effective in familiar environments such as participants’ homes (see Solano et al., 2020).

### Outlook

The key-press design employed in the current study appears to be a promising approach for assessing OGC more efficiently than in the previous mouse-tracking study (Landwehr et al., 2024). At the same time, concerns may remain that by focusing solely on decision outcomes—and omitting the underlying processes—diagnostically valuable information could be lost. In the previous study (Landwehr et al., 2024), we introduced a framework in which (1) misperception, (2) lack of self-control, and (3) egocentric hedonism were considered potential antecedents of OGC. We pointed out that by capturing participants’ mouse-cursor movements during a selection task (e.g., Freeman & Ambady, 2009), it might be possible to identify behavioral indicators of distinct mechanisms underlying the decision-making process (and thus potential risk factors indicated by an “unstable” decisional process inferred from the recorded mouse trajectories). For instance, it may happen that a woman’s dismissive attitude is indeed correctly perceived but knowingly and deliberately disregarded by an assessing male, especially since it has been shown that under certain conditions, approaching a social target is primarily driven by the target’s perceived utility for achieving the perceiver’s goals (Gruenfeld et al., 2008). We hypothesized that, within the context of a selection task, such a strong emphasis on one’s own sexual satisfaction (i.e., egocentric hedonism) would be reflected in relatively straight mouse trajectories, as the internal conflict in this case is low, whereas a lack of sexual self-control would result in more curved trajectories, reflecting the attempt to make the correct choice while resisting the attraction of a sexual distractor (Landwehr et al., 2024). Whether and to what extent diagnostic information is lost when using a mere key-press design—and thus whether the more technically and statistically demanding mouse-tracking procedure is warranted for forensic and clinical applications—remains to be determined by future comparative research. Also, as an alternative to returning to mouse-tracking or relying on isolated measurements of RT and ER, it might be possible to capture speed–accuracy interactions by meaningfully combining both measures into a single metric (Draheim et al., 2019).

As replicated in the present study, for a subgroup of male participants, sexual arousal appears to make it significantly more difficult to interpret and weight social cues in a stereotypically appropriate manner when these cues compete with cues that are perceived as more sexually appealing. An open question remains whether the changes in the response measures from T1 to T2, particularly in ER, resulted specifically from the induction of sexual arousal, or whether similar diagnostic changes—especially regarding correlations with external constructs of problematic sexuality—could also be observed under different conditions. For example, other forms of experimentally induced arousal might be interpreted in a context-dependent way, thereby aligning with an existing sexual goal (Schachter & Singer, 1962). Another possibility is that sexual arousal may interact with other sources of centrally processed excitation, as proposed in Toates’ (2009) hierarchical model of sexual motivation, with nonsexual arousal states potentially amplifying sexual arousal (e.g., anxiety; Bancroft, 1999). A control study involving different forms of arousal could also help clarify the present study’s unexpected result regarding the increase in errors from T2.1 to T2.2. Beyond broadening the scope of experimental conditions, future research would also benefit from expanding investigations to specific populations, both clinical (e.g., individuals with hypersexual tendencies) and forensic (e.g., sexual offenders).

### Conclusion

In an earlier study (Landwehr et al., 2024), we introduced an indirect measurement method to assess the tendency of heterosexual men to rely primarily on a woman’s global sexual cues rather than her specific affective cues when judging her sexual intent. The present conceptual replication aimed to replace the previously used mouse-tracking design with a simplified key-press procedure. All main and interaction effects essential for the functionality of the paradigm proved to be robust. In addition, meaningful correlations between the measured OGC indicators and external criteria related to problematic sexuality, assessed via self-reports, were again observed, even though the pattern of moderation by relevant sexual and nonsexual variables differed somewhat from previous findings. The intended simplification of the design through a streamlined experimental procedure can thus be considered successful, strengthening the expectation that the presented paradigm, with its behavioral approach, may be of diagnostic value in both forensic and clinical contexts. Furthermore, it is conceivable that a measurable individual tendency to base pre-approach sexual selection on hedonic, state-driven preferences rather than socially appropriate criteria could represent a relevant target for prevention-oriented interventions. Such interventions may aim to improve the ability to correctly recognize, interpret, and weight sexual and social cues, particularly in members of at-risk populations.

## Supplementary Information

Below is the link to the electronic supplementary material.

Supplementary file1 (DOCX 489 kb)

## Data Availability

Data and materials are available from the corresponding author upon reasonable request.

## Footnotes

1 Indeed, sexually disinhibiting effects of acute sexual arousal have also been demonstrated in women (Imhoff & Schmidt, 2014; Wiemer et al., 2023), albeit to a lesser extent, suggesting that such effects may constitute a human universal.

2 Although there are various motivations for flirting apart from initiating sexual contact, men often associate flirting cues with sexual intentions (Henningsen, 2004). Given that the present study specifically sought to measure heterosexual men’s responses to female flirting cues while in a state of sexual arousal, it focused on the prevailing male perception of flirting as indicative of sexual interest. Furthermore, in our flirting condition (see Stimuli and Trial Types section), the female models had been explicitly instructed to flirt with someone they found sexually attractive, i.e., to engage in sexually suggestive flirting.

3 The Sexual Inhibition Scale (SIS; Janssen et al., 2002) and the Acceptance of Modern Myths about Sexual Aggression Scale (AMMSA; Gerger et al., 2007), which had been used in the previous study (Landwehr et al., 2024), were not included in the present study, since (1) the SIS conceptually contrasts with the other scales and would therefore not contribute to a coherent interpretive framework across measures, and (2) rape-supportive attitudes, as assessed by the AMMSA, have already been extensively investigated in relation to cue recognition (e.g., Treat et al., 2017, 2020). For this reason, we prioritized limiting the number of items to maintain participant motivation in the present study.

4 The other two subscales of the SNS were not included because their constructs were either already covered by another scale (i.e., Sexual Exploitation; assessed by the SUSS) or considered less relevant to the hypothesized relationship with OGC (i.e., Grandiose Sense of Sexual Skill).

5 This procedure ensured that men who, at this point in the study, might have been more prone to errors in answering the manipulation-check questions due to heightened sexual arousal were not excluded because of overly complex items (i.e., to avoid false negatives). In addition, the manipulation checks confirmed that participants met the Prolific filter criterion of native-level German proficiency and had properly understood the instructions throughout the study.

6 While other essential preregistered aspects of the study (e.g., hypotheses, general design, exclusion criteria) were adhered to, the targeted sample size was not reached due to insufficient participant recruitment via Prolific. Consequently, data collection was terminated after 48 h without any new enrollments. To maintain sample homogeneity, we opted against expanding recruitment through a second source, especially as the sample size achieved was considered sufficient for the planned analyses to yield interpretable results.

7 This finding differed from expectations based on our initial study (Landwehr et al., 2024), in which trial construction had relied on a smaller-scale pre-validation indicating the opposite pattern of attractiveness ratings. This change in rating pattern may also partly be due to the fact that several dozen images from the original study had to be replaced due to copyright restrictions.

8 Notably, this revised operationalization may even be considered diagnostically more meaningful, as the hypothesized effects are tested under less polarized conditions, where selection should, in principle, be easier—thus making any observed response differences particularly telling and providing a stringent robustness check of earlier findings, while also increasing the study’s replication value through structural alignment with its predecessor. For details on how stimuli were selected for both trial-type conditions in this study, see ES3.

9 As preregistered, robustness checks were conducted using both more liberal and more conservative approaches to assess the stability of the observed effects. These analyses were performed on (1) the raw sample (*N* = 314) and (2) a more strictly filtered sample (*N* = 184) applying additional exclusion criteria—namely, participants who reported not being sexually aroused before T2 and those who answered more than one manipulation-check question incorrectly despite claiming to have listened to the audio narrative in full. In both cases, the overall substantive pattern of results remained stable across analyses.

10 Note that because smaller attractiveness differences indicate higher cue similarity, the negative correlations reflect higher ERs under greater similarity.

11 Note that positive *d*_z_ values indicate a decrease in the dependent variable from the earlier to the later phase (e.g., T2.1 > T2.2), whereas negative values indicate an increase.

12 Note that reported Δ*M* values for ER reflect absolute changes in percentage points rather than relative percentage changes.

13 To further ensure data quality, we implemented several direct and indirect manipulation checks (see Measures section) and preregistered conservative exclusion criteria (see Participants section).

## Code Availability

Analysis code is available from the corresponding author upon reasonable request.
