# Supplementary material for: A Dress Is (Still) Not a Yes: How Even Simple Key Presses Reveal Sexually Aroused Men’s Overreliance on Global Cues in the Context of Sexual Flirting
Source: Arch Sex Behav. 2026 Jun 1;55(4):1549–74. doi: 10.1007/s10508-026-03449-7 (PMC13275754; doi:10.1007/s10508-026-03449-7)

**Electronic Supplement**

**ES1: Theoretical Rationales for Moderator Selection**

**Sexual Narcissism**

Sexual narcissism is a domain-specific form of narcissism (Widman & McNulty, 2011) that manifests in sexual contexts and encompasses corresponding cognitive components, such as entitled sexual desires and a lack of empathy toward sexual partners (Widman & McNulty, 2010). The rationale for considering sexual narcissism as a moderator of the relationship between sexual arousal and OGC parallels that for sexual objectification, as both traits reflect an egocentric pattern of sexual motivation and behavior (Hurlbert et al., 1994). Individuals high in sexual narcissism view short-term sexual relationships as opportunities for self-affirmation (Gewirtz-Meydan, 2017), feel entitled to regular sexual activity (Widman & McNulty, 2010) and sexual satisfaction (Imhoff et al., 2013), and are willing to engage in manipulative courtship behavior (Klement et al., 2019). Due to their focus on self-gratification and higher levels of sexualization (Varga et al., 2022), it is likely that sexual narcissists disregard the opposing interests of a desired sexual partner by prioritizing sexual cues over conflicting affective ones. Moreover, narcissism is linked to low self-control and increased impulsivity, both of which indicate a preference for short-term rewards (Vazire & Funder, 2006) and may explain why sexually narcissistic individuals are more prone to infidelity (McNulty & Widman, 2014). Particularly relevant in the forensic context are findings linking sexual narcissism to problematic sexual attitudes and beliefs (e.g., Bouffard, 2010; Hill & Fischer, 2001) as well as to male-perpetrated sexual coercion and aggression (e.g., Basting et al., 2023). In this regard, sexually coercive behavior has been identified as a possible dysfunctional narcissistic response to unexpected rejection (Bushman et al., 2003).

**Dark Triad Traits**

The Dark Triad is a socially undesirable set of traits consisting of psychopathy, (global) narcissism, and Machiavellianism (Paulhus & Williams, 2002). Jones and Paulhus (2011, p. 249) defined psychopathy as “characterized by callousness, impulsive thrill-seeking, and criminal behavior,” narcissism as “associated with grandiosity, egocentrism, and a sense of personal entitlement,” and Machiavellianism as “marked by strategic manipulation.” The three constructs are conceptually distinct, with recent research indicating a significant overlap between Machiavellianism and psychopathy, whereas global narcissism has been identified as a separate construct (Rogoza & Cieciuch, 2018). Nevertheless, viewing the three traits collectively as “the malevolent aspects of human personality” (Wright et al., 2017) remains valid, with their common core being the tendency to manipulate and exploit others for selfish reasons and a general lack of empathy (e.g., Brewer et al., 2019; Jones & Figueredo, 2013). In the sexual domain, prior studies have indicated that Dark Triad traits contribute to manipulative and aggressive sexual behaviors in both men and women (e.g., Jones & Olderbak, 2014), such as manipulative seduction tactics aimed at persuading individuals who are initially reluctant to engage in sexual activity (Prusik et al., 2021), sexual coercion (Figueredo et al., 2015), sexual harassment (Zeigler-Hill et al., 2016), and sexual violence (Balcioglu et al., 2024). Particularly relevant in the context of the present study are findings suggesting that narcissism is indirectly linked to men’s misperception of women’s sexual intent (Wegner & Abbey, 2016).

**Propensity for Social Deviance**

Socially deviant behavior refers to actions that violate one or more normative rules of a social system, thereby contrasting with conforming behavior. Such norm violations can affect both individual social actors and social systems of various sizes and types (e.g., peer groups, workplace contexts, or society as a whole). A prototypical manifestation of socially deviant behavior is crime, i.e., the violation of legal norms (Cohen, 1959). Gottfredson and Hirschi (1990), in their studies on the origins of crime, noted that criminal acts allow for the rapid and effortless fulfillment of needs and are often committed by individuals with low self-control and a limited ability to delay gratification. Such individuals tend to react impulsively to tangible stimuli in their immediate environment, guided by a concrete “here and now” orientation (Hirschi, 2004). Although such behavior occurs in varying degrees of severity, it frequently involves a disregard for the interests of others in favor of self-centered gratification. In the present study, past socially deviant behavior was considered a moderator variable to examine whether a general tendency toward self-centered gratification would translate into an increased tendency toward OGC, i.e., a socially undesirable focus on preferred stimuli in a specifically sexual context.

**References**

Balcioglu, Y. H., Dogan, M., Incı, I., Tabo, A., & Solmaz, M. (2024). Understanding the dark side of personality in sex offenders considering the level of sexual violence. *Psychiatry, Psychology and Law*, *31*(2), 254–273. https://doi.org/10.1080/13218719.2023.2192259

Basting, E. J., Barrett, M. E., Garner, A. R., Florimbio, A. R., Sullivan, J. A., Medenblik, A. M., & Stuart, G. L. (2023). Sexual narcissism and hypersexuality relate to sexual coercion in hookups among U.S. university students. *Archives of Sexual Behavior*, *52*(6), 2577–2588. https://doi.org/10.1007/s10508-023-02580-z

Bouffard, L. A. (2010). Exploring the utility of entitlement in understanding sexual aggression. *Journal of Criminal Justice*, *38*(5), 870–879. https://doi.org/10.1016/j.jcrimjus.2010.06.002

Brewer, G., Lyons, M., Perry, A., & O’Brien, F. (2019). Dark Triad traits and perceptions of sexual harassment. *Journal of Interpersonal Violence*, *36*(13–14), NP7373–NP7387. https://doi.org/10.1177/0886260519827666

Bushman, B. J., Bonacci, A. M., van Dijk, M., & Baumeister, R. F. (2003). Narcissism, sexual refusal, and aggression: Testing a narcissistic reactance model of sexual coercion. *Journal of Personality and Social Psychology*, *84*(5), 1027–1040. https://doi.org/10.1037/0022-3514.84.5.1027

Cohen, A. K. (1959). The study of social disorganization and deviant behavior. In R. K. Merton, L. Broom, & L. S. Cottrell, Jr. (Eds.), *Sociology today: Problems and prospects* (pp. 461–484). Basic Books.

Figueredo, A. J., Gladden, P. R., Sisco, M. M., Patch, E. A., & Jones, D. N. (2015). The unholy trinity: The Dark Triad, sexual coercion, and Brunswik-symmetry. *Evolutionary Psychology*, *13*(2), 435–454. https://doi.org/10.1177/147470491501300208

Gewirtz-Meydan, A. (2017). Why do narcissistic individuals engage in sex? Exploring sexual motives as a mediator for sexual satisfaction and function. *Personality and Individual Differences*, *105*, 7–13. https://doi.org/10.1016/j.paid.2016.09.009

Gottfredson, M. R., & Hirschi, T. (1990). *A general theory of crime*. Stanford University Press.

Hill, M. S., & Fischer, A. R. (2001). Does entitlement mediate the link between masculinity and rape-related variables? *Journal of Counseling Psychology*, *48*(1), 39–50. https://doi.org/10.1037//0022-0167.48.1.39

Hirschi, T. (2004). Self-control and crime. In R. F. Baumeister & K. D. Vohs (Eds.), *Handbook of self-regulation: Research, theory, and applications* (pp. 537–552). Guilford Press.

Hurlbert, D. F., Apt, C., Gasar, S., Wilson, N. E., & Murphy, Y. (1994). Sexual narcissism: A validation study. *Journal of Sex & Marital Therapy*, *20*(1), 24–34. https://doi.org/10.1080/00926239408403414

Imhoff, R., Bergmann, X., Banse, R., & Schmidt, A. F. (2013). Exploring the automatic undercurrents of sexual narcissism: Individual differences in the sex-aggression link. *Archives of Sexual Behavior*, *42*(6), 1033–1041. https://doi.org/10.1007/s10508-012-0065-x

Jones, D. N., & Figueredo, A. J. (2013). The core of darkness: Uncovering the heart of the Dark Triad. *European Journal of Personality*, *27*(6), 521–531. https://doi.org/10.1002/per.1893

Jones, D. N., & Olderbak, S. G. (2014). The associations among dark personalities and sexual tactics across different scenarios. *Journal of Interpersonal Violence*, *29*(6), 1050–1070. https://doi.org/10.1177/0886260513506053

Jones, D. N., & Paulhus, D. L. (2011). Differentiating the Dark Triad within the interpersonal circumplex. In L. Horowitz & S. Strack (Eds.), *Handbook of interpersonal psychology: Theory, research, assessment, and therapeutic interventions* (pp. 249–267). Wiley. https://doi.org/10.1002/9781118001868.ch15

Klement, K. R., Xoxakos, P., Nazario, M., Erickson, J. M., Salley, S., Pieterick, M., DeLaFuente, J. L., Hemmerling, T. R., Martin, K., & Sagarin, B. J. (2019). Winning the game: How sexual narcissism relates to adversarial sexual beliefs and pick-up techniques. *Sexuality & Culture*, *23*(4), 1283–1300. https://doi.org/10.1007/s12119-019-09618-2

McNulty, J. K., & Widman, L. (2014). Sexual narcissism and infidelity in early marriage. *Archives of Sexual Behavior*, *43*(7), 1315–1325. https://doi.org/10.1007/s10508-014-0282-6

Paulhus, D. L., & Williams, K. M. (2002). The Dark Triad of personality: Narcissism, Machiavellianism, and psychopathy. *Journal of Research in Personality*, *36*(6), 556–563. https://doi.org/10.1016/s0092-6566(02)00505-6

Prusik, M., Konopka, K., & Kocur, D. (2021). Too many shades of gray: The Dark Triad and its linkage to coercive and coaxing tactics to obtain sex and the quality of romantic relationships. *Personality and Individual Differences*, *170*, Article 110413. https://doi.org/10.1016/j.paid.2020.110413

Rogoza, R., & Cieciuch, J. (2018). Dark Triad traits and their structure: An empirical approach. *Current Psychology*, *39*(4), 1287–1302. https://doi.org/10.1007/s12144-018-9834-6

Varga, B. A., Sal, D., Oosterhouse, L. B., Hevesi, K., & Rowland, D. L. (2022). Narcissism, sexual response, and sexual and relationship satisfaction. *Sexual and Relationship Therapy*, *39*(4), 1124–1144. https://doi.org/10.1080/14681994.2022.2073345

Vazire, S., & Funder, D. C. (2006). Impulsivity and the self-defeating behavior of narcissists. *Personality and Social Psychology Review*, *10*(2), 154–165. https://doi.org/10.1207/s15327957pspr1002_4

Wegner, R., & Abbey, A. (2016). Individual differences in men’s misperception of women’s sexual intent: Application and extension of the Confluence Model. *Personality and Individual Differences*, *94*, 16–20. https://doi.org/10.1016/j.paid.2015.12.027

Widman, L., & McNulty, J. K. (2010). Sexual narcissism and the perpetration of sexual aggression. *Archives of Sexual Behavior*, *39*(4), 926–939. https://doi.org/10.1007/s10508-008-9461-7

Widman, L., & McNulty, J. K. (2011). Narcissism and sexuality. In W. K. Campbell & J. D. Miller (Eds.), *The handbook of narcissism and narcissistic personality disorder: Theoretical approaches, empirical findings, and treatments* (pp. 351–359). Wiley. https://doi.org/10.1002/9781118093108.ch31

Wright, J. P., Morgan, M. A., Almeida, P. R., Almosaed, N. F., Moghrabi, S. S., & Bashatah, F. S. (2017). Malevolent forces: Self-control, the Dark Triad, and crime. *Youth Violence and Juvenile Justice*, *15*(2), 191–215. https://doi.org/10.1177/1541204016667995

Zeigler-Hill, V., Besser, A., Morag, J., & Keith Campbell, W. (2016). The Dark Triad and sexual harassment proclivity. *Personality and Individual Differences*, *89*, 47–54. https://doi.org/10.1016/j.paid.2015.09.048

**ES2: Conception, Creation, and Validation of the Stimuli**

For a detailed description of the conception, creation, and validation of the PICSA database, see the corresponding preprint:

https://doi.org/10.31234/osf.io/b3629_v1

The preprint provides a full account of the methodological background, stimulus creation, rating procedure, and validation studies.

**ES3: Stimulus Properties and Parallel Set Formation in the Current Study**

To maximize the diagnostic value of the present paradigm and assess the robustness of earlier findings (Landwehr et al., 2024), image pairs selected for the high-conflict (HC) trial condition in the current study were required to feature a stereotypically correct image—displaying the Casual × Flirting (CaFl) cue combination—that exhibited equal or higher sexual attractiveness than the stereotypically incorrect image—displaying the Sexy × Rejecting (SeRe) cue combination. For this purpose, a sexual-attractiveness difference score was calculated for each HC image pair (CaFl − SeRe) based on normative ratings collected in a prior validation study (Landwehr & Landwehr, 2025). Only HC pairs with a difference score ≥ 0 were included in the experimental set, ensuring that the correct image was perceived as at least equally, and typically more, sexually attractive, with values close to zero marking the lower bound of the selected range. This selection logic served two purposes: (1) to ensure conceptual consistency across all HC trials, and (2) to create a gradient of cue similarity reflecting trial difficulty within the HC condition. Specifically, trials with highly similar attractiveness values (i.e., smaller difference scores) were assumed to elicit greater selection conflict, thereby potentially increasing RTs and ERs—especially in individuals with a tendency toward OGC, regardless of the underlying mechanism (Landwehr et al., 2024).

Based on the validation study by Landwehr and Landwehr (2025), in which all images had been evaluated on multiple dimensions using a 7-point scale by *N* = 150 raters from an age-stratified sample, 52 HC image pairs met the selection criterion described above. The pairwise difference scores of these image pairs ranged from 0.02 to 1.18 (*M* = 0.42, *SD* = 0.26). In the next step, pairwise mean sexual-attractiveness values were calculated for the 52 HC pairs, ranging from 1.99 to 5.20 (*M* = 3.57, *SD* = 0.81). As expected, a *t* test conducted on the selected HC set (CaFl vs. SeRe) yielded a clear result, *t*(51) = 11.94, *p* < .001, *d*_z_ = 1.66, indicating a distinct group difference, with images displaying the CaFl combination being rated significantly higher in sexual attractiveness.

To complement the selection logic of the HC condition, image pairs selected for the low-conflict (LC) trial condition were required to feature a stereotypically correct image—displaying the Sexy × Flirting (SeFl) cue combination—that exhibited higher sexual attractiveness than the stereotypically incorrect image—displaying the Casual × Rejecting (CaRe) cue combination. As in the HC condition, a sexual-attractiveness difference score was calculated for each LC image pair (SeFl − CaRe) based on normative ratings from the full pictorial stimulus set (Landwehr & Landwehr, 2025). To ensure low decision conflict across the LC condition, only image pairs with a clearly positive difference score were included in the experimental set. Specifically, the 52 image pairs with the highest difference scores were selected to match the number of trials across conditions, thereby creating a consistently low-conflict baseline for comparison with the HC condition. The pairwise difference scores of these LC pairs ranged from 0.56 to 1.66 (*M* = 1.00, *SD* = 0.29), and their pairwise mean sexual-attractiveness values ranged from 2.25 to 5.26 (*M* = 3.66, *SD* = 0.72). As anticipated, a *t* test conducted on the selected LC set (SeFl vs. CaRe) yielded an unambiguous result, *t*(51) = 24.55, *p* < .001, *d*_z_ = 3.40, confirming the expected group difference, with images displaying the SeFl combination being rated significantly higher in sexual attractiveness.

To ensure comparability between the two measurements, two parallel stimulus sets were constructed from the 2 × 52 selected image pairs using the same multi-step procedure as in our previous study (Landwehr et al., 2024). Specifically, the image pairs were allocated to two subsets (Set A and Set B) and iteratively balanced based on the following criteria: (1) mean pairwise difference scores, (2) distribution and range of pairwise difference scores, (3) mean pairwise sexual-attractiveness values, and (4) distribution and range of pairwise sexual-attractiveness values. For full details, see Tables ES1 and ES2 as well as Figures ES3 and ES4 (visualization of set matching). The final parallel sets were composed such that the first set included the A subsets from both trial conditions and the second set included the B subsets.

**References**

Landwehr, I., & Landwehr, K. (2025). *PICSA: A pictorial stimulus set of (in)congruent sexual and affective cues in women*. PsyArXiv. https://doi.org/10.31234/osf.io/b3629_v1

Landwehr, I., Mundloch, K., & Schmidt, A. F. (2024). A dress is not a yes: Towards an indirect mouse-tracking measure of men’s overreliance on global cues in the context of sexual flirting. *Archives of Sexual Behavior*, *53*(6), 2063–2082. https://doi.org/10.1007/s10508-023-02798-x

**Table ES1**

*Mean Difference Scores and Mean Sexual Attractiveness Values of the Subsets*

|  | High-Conflict Condition | |  | Low-Conflict Condition | |
| --- | --- | --- | --- | --- | --- |
|  | Difference | Attractiveness |  | Difference | Attractiveness |
| Subset A | 0.41 (0.23) | 3.57 (0.78) |  | 1.02 (0.29) | 3.66 (0.74) |
| Subset B | 0.43 (0.27) | 3.58 (0.85) |  | 0.98 (0.30) | 3.66 (0.70) |

*Note*. Standard deviations in brackets.

**Table ES2**

*Ranges of Pairwise Differences Scores and Pairwise Mean Sexual Attractiveness Values in the Subsets*

|  | High-Conflict Condition | |  | Low-Conflict Condition | |
| --- | --- | --- | --- | --- | --- |
|  | Differences | Attractiveness |  | Differences | Attractiveness |
| Subset A | [0.04; 0.88] | [2.13; 4.98] |  | [0.62; 1.66] | [2.25; 5.26] |
| Subset B | [0.02; 1.18] | [1.99; 5.20] |  | [0.56; 1.64] | [2.26; 4.87] |

**Figure ES1**

*Moderation of the Socially Undesirable Sexual Selection Scale (SUSS) Score (Mean-Centered) on the Relationship Between Sexual Arousal and Overreliance on Global Cues, as Indicated by Error Rates*


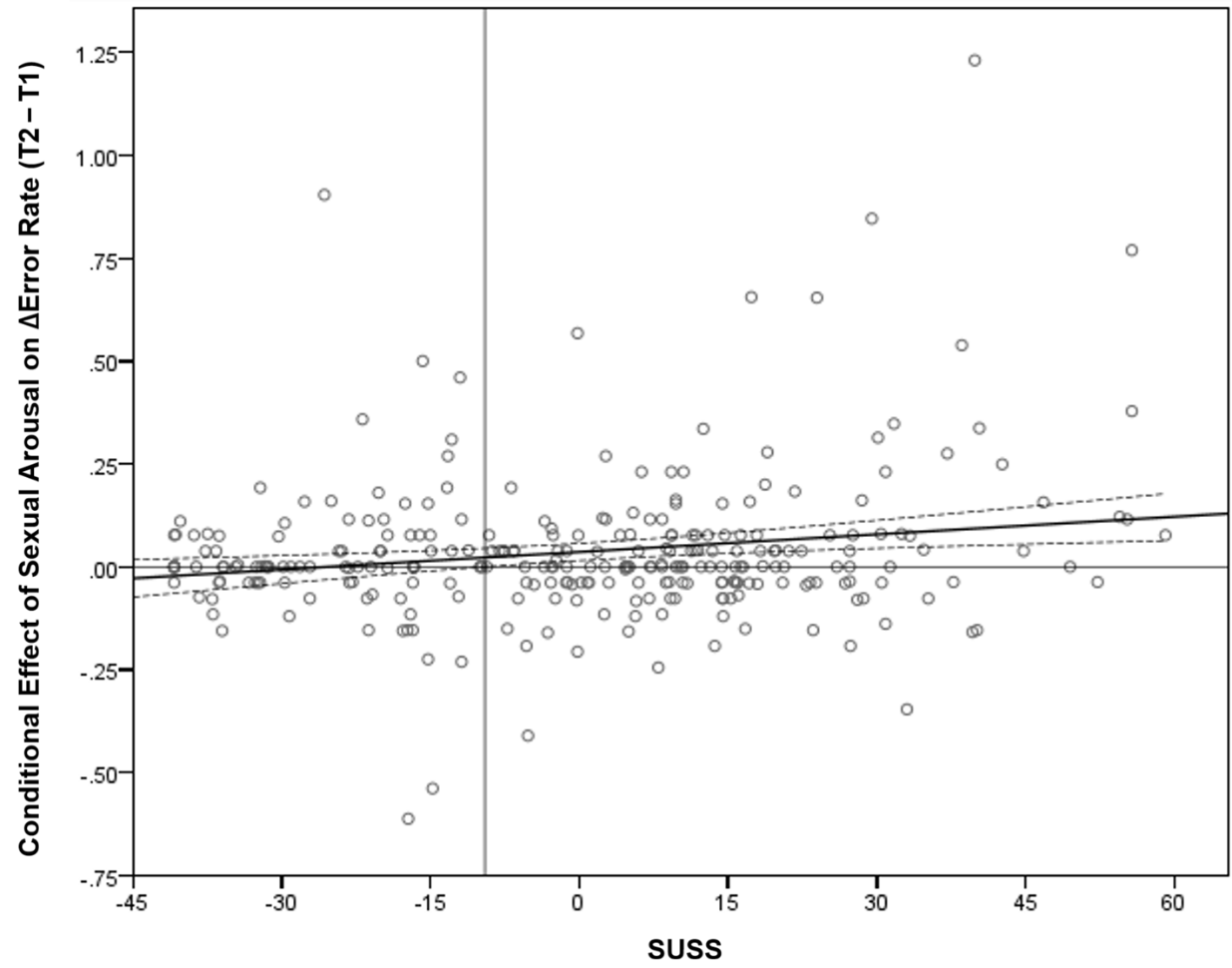


*Note*. The vertical line denotes the Johnson-Neyman criterion, marking the point on the X-axis beyond which the moderator has a significant influence on the dependent variable (i.e., where the lower limit of the 95% confidence interval exceeds 0; here: 64% of the sample).

**Figure ES2**

*Moderation of the Sexual Manipulativeness, Exploitativeness, and Disinhibition (SMED) Factor (Mean-Centered) on the Relationship Between Sexual Arousal and Overreliance on Global Cues, as Indicated by Error Rates*


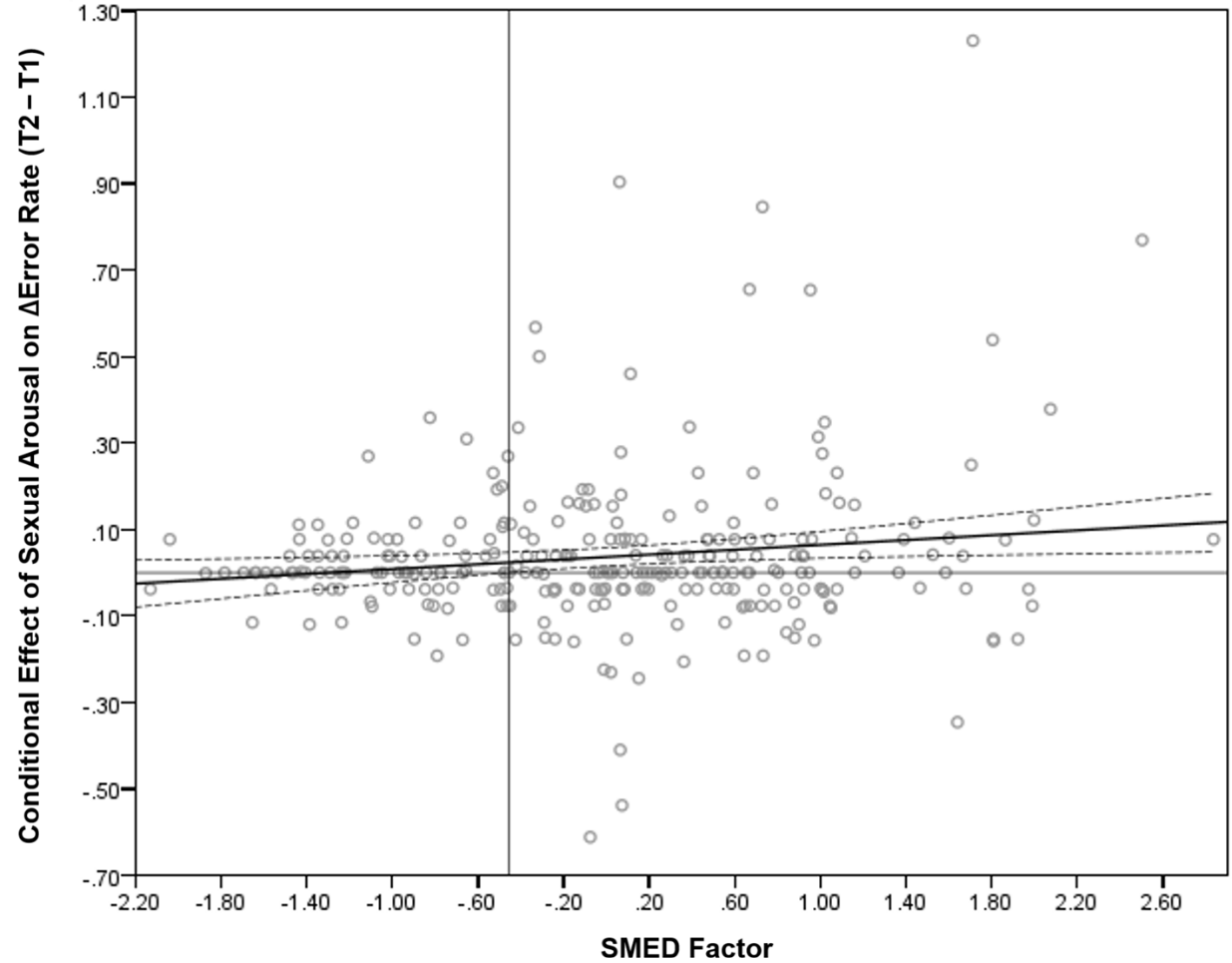


*Note*. The vertical line denotes the Johnson-Neyman criterion, marking the point on the X-axis beyond which the moderator has a significant influence on the dependent variable (i.e., where the lower limit of the 95% confidence interval exceeds 0; here: 68% of the sample).

**Figure ES3**

*Distribution of Pairwise Difference Scores Across Both Trial Conditions*


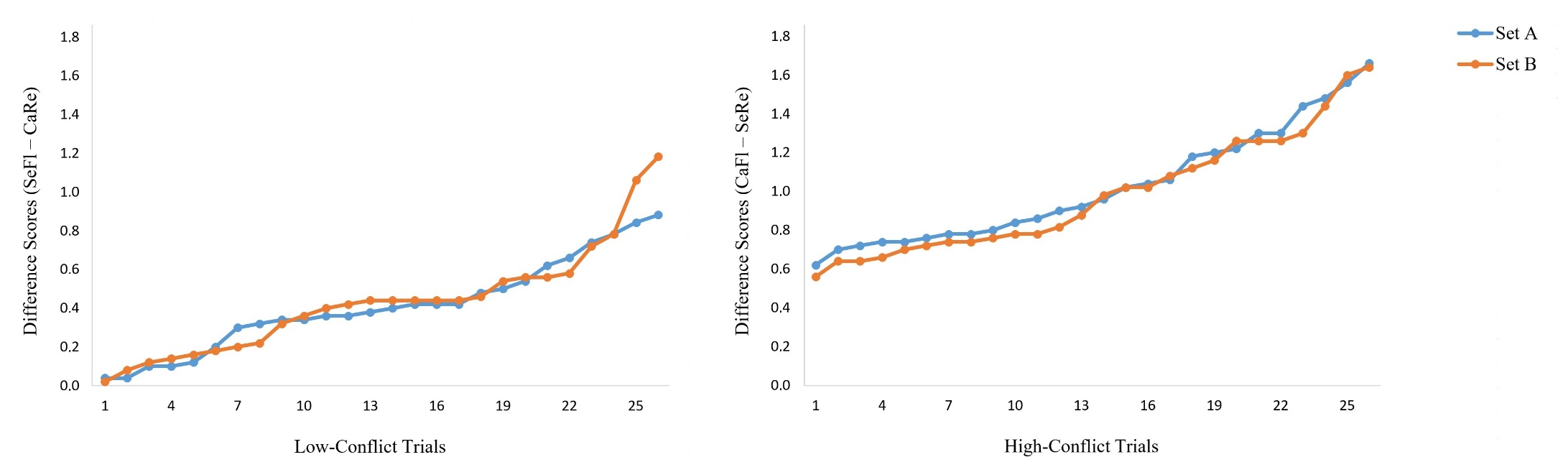


**Figure ES4**

*Distribution of Pairwise Mean Sexual-Attractiveness Values Across Both Trial Conditions*


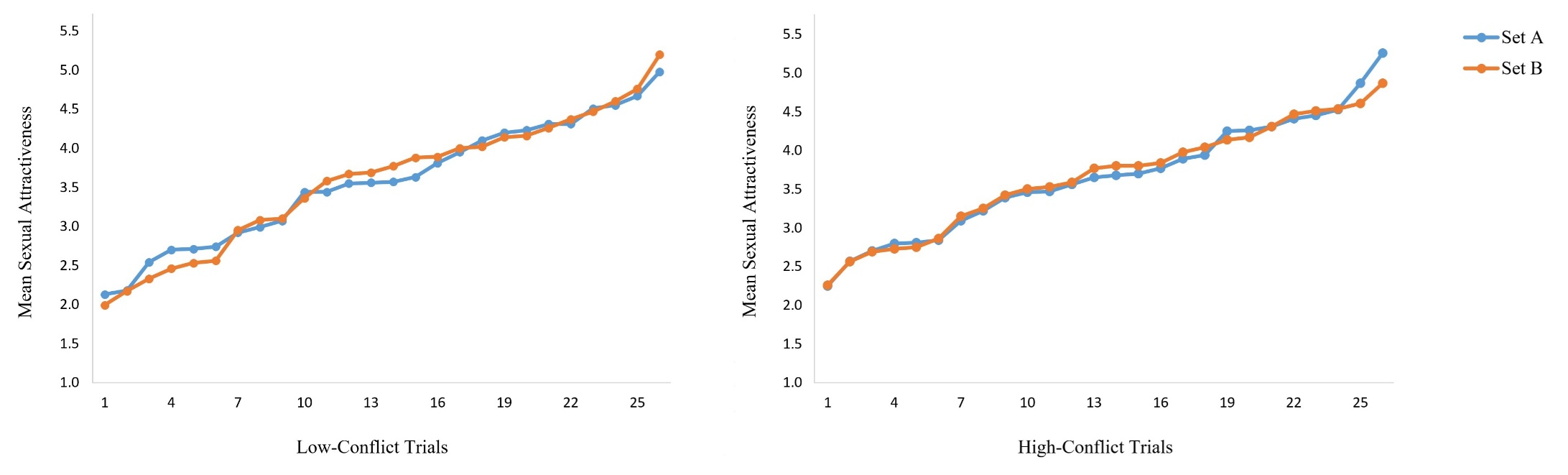

Supplement: Supplementary file 1 — Supplementary file1 (DOCX 489 kb) [file 10508_2026_3449_MOESM1_ESM.docx]
